# 25-Hydroxyvitamin D reference percentiles and the role of their determinants among European children and adolescents

**DOI:** 10.1038/s41430-021-00985-4

**Published:** 2021-07-23

**Authors:** Maike Wolters, Timm Intemann, Paola Russo, Luis A. Moreno, Dénes Molnár, Toomas Veidebaum, Michael Tornaritis, Stefaan De Henauw, Gabriele Eiben, Wolfgang Ahrens, Anna Floegel

**Affiliations:** 1 Leibniz Institute for Prevention Research and Epidemiology—BIPS, Bremen, Germany; 2 Institute of Food Sciences, National Research Council, Avellino, Italy; 3 GENUD (Growth, Exercise, Nutrition and Development) Research Group, Faculty of Health Sciences, Universidad de Zaragoza, Instituto Agroalimentario de Aragón (IA2), Instituto de Investigación Sanitaria Aragón (IIS Aragón), Zaragoza, Spain and Centro de Investigación Biomédica en Red de Fisiopatología de la Obesidad y Nutrición (CIBERObn), Instituto de Salud Carlos III, Madrid, Spain; 4 Department of Pediatrics, Medical School, University of Pécs, Pécs, Hungary; 5 National Institute for Health Development, Estonian Centre of Behavioral and Health Sciences, Tallinn, Estonia; 6 Research and Education Institute of Child Health, Strovolos, Cyprus; 7 Department of Public Health and Primary Care, Ghent University, Ghent, Belgium; 8 Department of Public Health and Community Medicine, University of Gothenburg, Gothenburg, Sweden; 9 Department of Public Health, School of Health Sciences, University of Skövde, Skövde, Sweden; 10 Institute of Statistics, Faculty of Mathematics and Computer Science, University of Bremen, Bremen, Germany

**Keywords:** Biomarkers, Nutrition

## Abstract

**Background/objectives:**

To provide age- and sex-specific percentile curves of serum 25-hydroxyvitamin D (25(OH)D) by determinants from 3-<15 year-old European children, and to analyse how modifiable determinants influence 25(OH)D.

**Subjects/methods:**

Serum samples were collected from children of eight European countries participating in the multicenter IDEFICS/I.Family cohort studies. Serum 25(OH)D concentrations were analysed in a central lab by a chemiluminescence assay and the values from 2171 children (*N* = 3606 measurements) were used to estimate percentile curves using the generalized additive model for location, scale and shape. The association of 25(OH)D with time spent outdoors was investigated considering sex, age, country, parental education, BMI *z* score, UV radiation, and dietary vitamin D in regressions models.

**Results:**

The age- and sex-specific 5th and 95th percentiles of 25(OH)D ranged from 16.5 to 73.3 and 20.8 to 79.3 nmol/l in girls and boys, respectively. A total of 63% had deficient (<50 nmol/l), 33% insufficient (50-<75 nmol/l) and 3% sufficient (≥75 nmol/l) levels. 25(OH)D increased with increasing UV radiation, time spent outdoors, and vitamin D intake and slightly decreased with increasing BMI *z* score and age. The odds ratio (OR) for a non-deficient 25(OH)D status (reference category: deficient status) by one additional hour spent outdoors was 1.21, 95% CI [1.12–1.31], i.e., children who spent one more hour per day outdoors than other children had a 21% higher chance of a non-deficient than a deficient status.

**Conclusion:**

A majority of children suffer from deficient 25(OH)D. UV radiation, outdoor time, and dietary vitamin D are important determinants of 25(OH)D.

## Introduction

Lack of sunlight has been associated with rickets in children as early as the 19th century. Rickets, a bone-deforming disease, is caused by severe vitamin D deficiency and results in growth retardation, muscle weakness and skeletal abnormalities [1]. It was widespread among children who lived in the crowded and polluted cities of Northern Europe during the industrialization. Ever since, the encouragement of sensible sun exposure, vitamin D supplementation in infants, and vitamin D fortification of milk almost eradicated the disease in industrialized countries. However, vitamin D deficiency and even rickets are again emerging in children of the high income countries [1]. Vitamin D deficiency leads to low bone mineral density and increases fracture risk [2]. In addition, it has been associated with various non-skeletal diseases such as increased risk of autoimmune diseases, infectious diseases, cardiovascular diseases and common cancers as well as all-cause mortality [2–6]. A matter of discussion remains whether vitamin D is causal for the association with non-skeletal diseases or rather a marker of poor health [3, 4]. Vitamin D is produced in the skin by exposure to ultraviolet (UV)-B radiation, or taken up from food or supplements. It is hepatically metabolized to 25-hydroxyvitamin D (25(OH)D) which is most commonly used to determine vitamin D status [5, 7] and usually categorized as deficient (<50 nmol/l), insufficient (50-<75 nmol/l) or sufficient (≥75 nmol/l) based on optimal calcium absorption efficiency [5, 8, 9]. Results of previous European studies showed that approximately 80% of children and adolescents had insufficient or even deficient vitamin D levels [10, 11]. However, data on the vitamin D status of young children are limited. In a small sample of Belgian children aged 3–11 years, vitamin D status was insufficient in 53% and deficient in 5% of the children [12]. Yet, large studies on vitamin D status are lacking in European children [13]. Besides, vitamin D concentrations have been reported to be lower in children and adolescents with obesity than in normal weight [10, 14] and to underlie seasonal influences due to the production in the skin by UV-B radiation [15]. That is also why living at higher latitudes increases the risk of vitamin D deficiency [10]. In the Belgian IDEFICS children, month of blood draw, weekly number of hours playing outside and body composition were previously identified as important determinants of vitamin D status [12]. To sum up, data are rare on vitamin D status of European children, particularly in a large sample of children and adolescents, across a wide age range and considering different countries and their specific UV radiation. Furthermore, few studies have differentiated vitamin D status between children with normal weight, overweight and obesity.

The aim of this study was therefore to establish sex-specific percentile curves of 25(OH)D concentrations as biomarker of the vitamin D status by age, body mass index (BMI) *z* score, weight-to-height ratio, time spent outdoors, dietary vitamin D intake, and UV radiation of 3 to 15-year-old European children. The generalized additive model for location, scale and shape [16] was used to derive percentile curves. Further, the prevalence of vitamin D deficiency and insufficiency was assessed. In regression analyses, the association of the 25(OH)D concentration with time spent outdoors was assessed considering age, BMI *z* score, dietary vitamin D intake, country of residence and cloud-modified UV radiation at the place of residence during the second last month before blood draw.

## Materials/subjects and methods

### Study population and data

The IDEFICS (Identification and Prevention of Dietary- and Lifestyle-Induced Health Effects in Children and Infants)/I.Family cohort is a pan-European population-based study which aimed to investigate the role of diet and lifestyle and their determinants in the development of health and disease during childhood and adolescence (ISRCTN registry, no. 62310987). In the baseline examination wave (Wave 1 = T0, 2007/2008) which was conducted in eight European countries (Belgium, Cyprus, Estonia, Germany, Hungary, Italy, Spain, and Sweden) a total of 16,229 children aged 2–9 years participated. Follow-up examinations were conducted in 2009/2010 (Wave 2 = T1) including 13,596 children and in 2013/2014 (Wave 3 = T3), where 7105 of the children participating already in T0 and/or T1 and 2512 newly recruited children were included. T0 and T3 data were used in this study. During all survey waves the same standardized assessments and procedures were applied which included physical examinations of the children as well as the collection of blood samples, questionnaires concerning lifestyle habits and dietary intakes completed by the children aged 12 years and older and by the parents for their children aged up to 12 years. A computer-assisted 24-hour dietary recall (24HDR) was used to assess the dietary intake of the children [17–19]. Information on diseases and use of drugs and nutrient supplements was collected in an interview with a parent. Detailed information on the design and objectives of the IDEFICS/I.Family studies can be found in Ahrens et al. [20, 21].

Parents provided written informed consent before children entered the studies. In addition, written consent was obtained from children aged 12 years and older, while oral consent for the examinations and sample collection was obtained from younger children. The institutional review boards of all eight study centers gave ethical approval for the studies.

### Serum 25-hydroxyvitamin D concentration

Fasting blood samples were drawn by venipuncture in the morning. Blood collection, processing, shipment and storage were conducted according to a quality management system for the collection of biological samples [22]. The serum was immediately separated in the study center and shipped on dry ice to Bremen, Germany, where it was stored in the BIPS biobank at −80 °C until analysis. 25(OH)D concentrations were measured in a central lab at the Institute of Clinical Chemistry and Laboratory Medicine in Greifswald, Germany, by the chemiluminescence assay, IDS-iSYS 25-Hydroxy Vitamin Dˢ (Immunodiagnostic Systems Ltd, Boldon, United Kingdom). According to the information of the manufacturer, the assay is aligned to the National Institute of Standards and Technology Standard Reference Material, (NIST SRM) 2972 (NIST, Gaithersburg, Maryland, USA) and covers a measurement range of 10–275 nmol/l (4–110 ng/ml).

The following categories were used to characterize the vitamin status: deficient (<50 nmol/l), insufficient (50-<75 nmol/l) or sufficient (≥75 nmol/l) [5, 8, 9]. The two latter are also categorized as non-deficient.

### Determinants of vitamin D status and covariates

Anthropometric measures: body weight was assessed to the nearest 0.1 kg in children wearing only light underwear in fasting state on a calibrated scale (adapted Tanita BC 420 MA for children up to six years, BC 418 MA for children >6 years, Tanita Europe GmbH, Sindelfingen, Germany). Height was measured to the nearest 0.1 cm with a calibrated stadiometer (Seca 225/213 stadiometer, Birmingham, UK). BMI was calculated as weight [kg] divided by height [m] squared. Waist circumference was measured to the nearest 0.1 cm in upright position with relaxed abdomen, midway between the lowest rib margin and the iliac crest. Waist-to-height ratios were calculated as waist [m] divided by height [m]. BMI *z* scores were determined based on Cole & Lobstein [23].

Skin exposure to sunlight is required for endogenous vitamin D synthesis and is thus associated with higher serum 25(OH)D concentrations [24]. UV-B radiation exposure depends on the UV radiation at the place of residence at a given time and on the time spent outdoors. UV dose data from the Tropospheric Emission Monitoring Internet Service (TEMIS, http://www.temis.nl/uvradiation/) were used which provides time series of UV dose data derived from atmospheric satellite data in the form of HDF-4 files for selected locations since 2004. As these data were available only for two of our study centers (Tallinn, Nicosia), we used the UV dose data of cities located close to the other study centers, in the same country and at similar latitude [25], which in turn were located close to the residence of the participating children (Table 1). The TEMIS database provides UV radiation data with several adjustments including that for the cloud cover calculated from METEOSAT data. Based on these data, more reliable information about the real UV dose on the earth’s surface than that of the cloud-free UV dose is available. In our analyses, we have used the place-, year-, and month-specific cloud-modified vitamin-D UV dose (UVDVC) expressed in kJ/m^2^ [26, 27].

**Table 1:** Places with available cloud-modified UV dose information* used as reference for the study center locations.

Country	Reference city with UV dose data	Latitude*	IDEFICS/I.Family study center	Latitude^#^
Estonia	Tallinn	59.4°N	Tallinn Tartu	59.4°N 58.4°N
Sweden	Norrkoping	58.6°N	Gothenburg	57.7°N
Belgium	Uccle	50.8°N	Ghent	51.1°N
Germany	Berlin	52.5°N	Wilhelmshaven Delmenhorst	53.5°N 53.1°N
Hungary	Budapest	47.5°N	Pecs Zalaegerszeg	46.1°N 46.8°N
Italy	Rome	41.9°N	Avellino	40.9°N
Spain	Madrid	40.4°N	Zaragoza	41.7°N
Cyprus	Nicosia	35.2°N	Nicosia Paphos	35.2°N 34.7°N

*https://www.temis.nl/uvradiation/UVarchive/stations_uv.php

^#^https://www.latlong.net/

The half-life of serum 25(OH)D is around 15–30 days [28, 29] and the 25(OH)D concentration reflects the UV exposure of the previous 4–8 weeks [26, 29, 30]. As the UVDVC of the second last month before blood draw showed a higher correlation (*r* = 0.39, 95% CI [0.36, 0.42]) with 25(OH)D than the month before blood sampling (*r* = 0.30, 95% CI [0.27, 0.33]), for each child the UVDVC of the second last month before the month of blood sampling at the respective place and survey wave was taken. In the following, “UVDVC” means the UVDVC value of the second last month before blood draw at (or near) the place of the residence and thus reflects the true UV dose more accurately than just the season as used in most previous studies.

The usual time spent outdoors was calculated based on the assessment by a self-administered parental questionnaire in young children up to 11 years (T0) and a self-administered questionnaire in older children (number of hours playing or “hanging out” outdoors during weekdays and weekend days). Dietary vitamin D intake (µg/d) was calculated based on the 24HDR and applying the German food composition table (German Nutrient Data Base, BLS II.3.1) [31] for the recalls of all included countries.

The highest education level of the parents according to the International Standard Classification of Education (ISCED) [32] was grouped in three categories: low (ISCED 0, 1, 2); medium (ISCED 3, 4) and high level (ISCED 5, 6).

### Analysis dataset

**Fig. 1:**
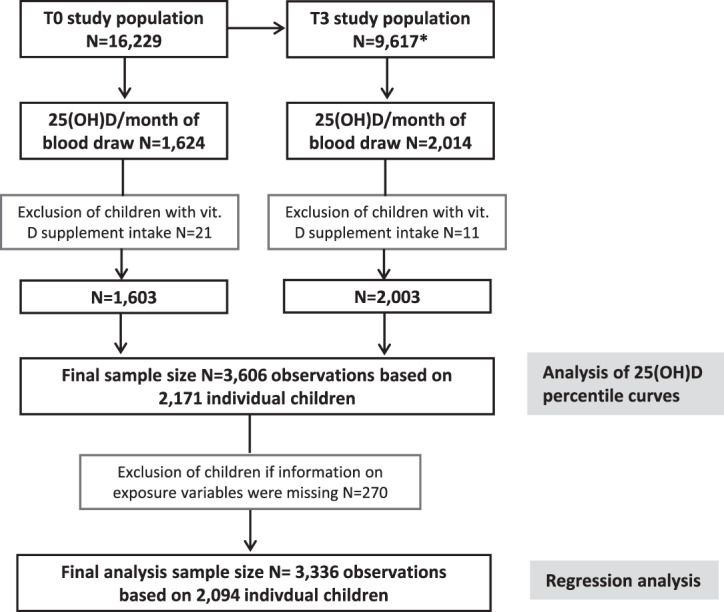
Flow chart of the children included in the analyses. *Including 1050 newly recruited children at T1 and 1512 newly recruited children at T3. T0, baseline survey; T1, follow-up after two years; T3, follow-up after six years; 25(OH)D 25-hydroxyvitamin D; ISCED international standard classification of education; vit. vitamin.

Laboratory measurements of 25(OH)D and dates of blood draw were available for *N* = 1624 measurements at T0 and *N* = 2014 at T3. Children who took vitamin D supplements were excluded which resulted in *N* = 1603 measurements at T0 and *N* = 2003 at T3. As the data of *N* = 1435 children were included at both time points, the final dataset included *N* = 2171 children (*N* = 3606 measurements) for the analysis of the percentile curves. For the regression analysis, *N* = 270 measurements (77 children) had to be excluded because of missing information on exposures (ISCED, time spent outdoors), thus, a total *N* = 3336 measurements based on 2094 individual children were included (Fig. 1).

### Descriptive statistic of study population

We calculated the mean and the standard deviation (SD) of all used continuous variables and the absolute numbers and percentages of all categorical variables for the entire study population and separately for T0 and T3. Furthermore, the mean and SD of 25(OH) D was calculated by sex, country, weight status category and ISCED. For children with available measurements of 25(OH)D at T0 and T3, we calculated the corresponding correlation coefficient. For this sample, we also created a contingency table to compare the 25(OH)D categories between T0 and T3.

### Estimation of usual vitamin D intake

After calculating the daily vitamin D intake for the repeated recalls with energy of at least 500 kcal, we applied the so-called NCI-method [33] to estimate the usual vitamin D intake (*N* = 2508). The method takes the skewness of the intake distribution into account and corrects for variance inflation caused by daily variation in diet, which leads to biased effect estimates. The usual vitamin D intake was estimated separately for T0 and T3 as well as for boys and girls. Age, BMI *z* score and the sequence of days were considered as covariates. An average of 1.4 and 2.1 recall days were available per child for estimation in T0 and T3, respectively. For this part of the analysis we used the statistical software SAS 9.3.

### Reference curve estimation

We used the generalized additive models for location, scale and shape (GAMLSS) [34] to estimate sex-specific reference curves of 25(OH)D depending separately on age, BMI *z* score, time spent outdoors, usual vitamin D intake and UVDVC. Furthermore, a model considering all of these covariates simultaneously was considered. We investigated models with different distributions (normal, Box-Cox Cole, Box-Cox *t* and Box-Cox power exponential distribution) with distribution parameters depending on constant, linear or P-spline functions of the covariates. The goodness of fit of the sex-specific models was assessed by the Bayesian Information Criterion and worm plots (For details regarding model selection see [35]). The final selected models are listed in Supplementary Table 1.

### Association analysis

A mixed effects logistic regressions model with the outcome non-deficient 25(OH)D status (yes/no) and the covariates time spent outdoors, age, BMI *z* score, UVDVC of the second last month before blood draw, sex, country, and ISCED considering a random effect term for repeated measurements were fitted to the data (main model). Furthermore, for sensitivity analysis additional exposures were used as covariates to fit three other models: [i] an interaction term of UVDVC and time spent outdoors to account for their joint effect, [ii] usual vitamin D intake, [iii] both of [i] and [ii].

Corresponding to the main model, a linear mixed model was fitted to investigate the association of the continuous 25(OH)D concentrations with time spent outdoors, adjusted for the same variables as in the mixed logistic regression model. A corresponding sensitivity analysis was also carried out.

We calculated odds ratio and β estimates and estimated the corresponding 95% confidence intervals using the profile likelihood method. For this part of the analysis we used the package lme4 [36] of the statistical software R 3.6.2 [37].

## Results

Table 2 shows the characteristics of the analysis group stratified by examination wave (T0 and T3). The mean 25(OH)D concentration was 45.2 (SD 16.7) nmol/l. Serum 25(OH)D levels were deficient in 63% of the children, insufficient in 33% and sufficient in only 3%. A high proportion of children (*N* = 914; 64%) were still in the same vitamin D status category in T3 as in T0 (Supplementary Table 2). In line with this, 25(OH)D concentrations were correlated between T0 and T3 (*r* = 0.47, 95% CI [0.43, 0.51]). Mean usual vitamin D intake was low (1.7 µg/d). The BMI *z* score was much higher at T3 than at T0. Accordingly, 15% of the children were classified as overweight or obese at T0 while this applied for 23% at T3. The time spent outdoors was much higher at T0 than at T3, i.e., 2.41 versus 1.80 h per day. Mean 25(OH)D was lower in girls than in boys and lower in Northern than in Southern European countries with two exceptions (Table 3). While the mean 25(OH)D level of children from Sweden was similar to Hungary and Spain, the mean 25(OH)D level of children from Italy was much lower and lay in the range of the children from Germany and Estonia. The mean 25(OH)D concentrations were lower for higher weight status and higher with higher parental education. The average UVDVC mostly reflected the latitude of the locations (Table 3).

**Table 2:** Characteristics of the total study population and the population separated by survey wave: T0, baseline. T3, 6-year follow-up.

Variables		Mean	SD	T0: Mean	T0: SD	T3: Mean	T3: SD
Age	years	9.4	3.4	6.2	1.8	12.0	1.8
BMI-z-score*		0.32	1.08	0.17	1.07	0.45	1.07
Waist-to-height-ratio		0.44	0.05	0.46	0.04	0.43	0.05
UVDVC	kJ/m^2^	1.8	2.0	1.6	2.0	2.0	2.0
Time spent outdoors	h/day	2.08	1.37	2.41	1.39	1.80	1.29
Vitamin D intake	µg/d	1.7	0.5	1.5	0.2	1.9	0.6
25-hydroxyvitamin D	nmol/l	45.2	16.7	44.7	15.4	45.6	17.8
		***N***	**%**	**T0:** ***N***	**T0: %**	**T3:** ***N***	**T3: %**
25-hydroxyvitamin D categories^#^	<50 nmol/l	2275	63	998	62	1277	64
	50–75 nmol/l	1208	33	562	35	646	32
	≥75 nmol/l	123	3	43	3	80	4
Sex	Boys	1855	51	823	51	1032	52
	Girls	1751	49	780	49	971	48
Country	Italy	353	10	94	6	259	13
	Estonia	603	17	286	18	317	16
	Cyprus	66	2	37	2	29	1
	Belgium	205	6	95	6	110	5
	Sweden	461	13	218	14	243	12
	Germany	560	16	216	13	344	17
	Hungary	826	23	389	24	437	22
	Spain	532	15	268	17	264	13
Weight status categories*	underweight	356	10	196	12	160	8
	normal weight	2564	71	1175	73	1389	69
	overweight	516	14	174	11	342	17
	obese	170	5	58	4	112	6
ISCED	low	138	4	51	3	87	4
	middle	1537	43	671	43	866	44
	high	1877	53	849	54	1028	52

*ISCED* international standard classification of education, *UVDVC* cloud-modified vitamin D UV dose of the second last month before blood draw

*Cole and Lobstein 2012 [23].

^#^deficient (<50 nmol/l), insufficient (50-<75 nmol/l), sufficient (≥75 nmol/l).

**Table 3:** Mean 25-hydroxyvitamin D by sex, country, weight status and education level of the parents (ISCED) and average UVDC by country.

Variables		Mean 25(OH)D (nmol/l)	SD 25(OH)D (nmol/l)	average UVDVC (kJ/m^2^)	SD average UVDVC (kJ/m^2^)
Sex	Boys	46.1	16.9		
	Girls	44.3	16.5		
Country	Italy	38.5	12.8	2.5	2.1
	Estonia	38.0	14.2	0.6	0.9
	Cyprus	53.0	12.5	4.1	2.6
	Belgium	45.0	12.7	1.0	1.2
	Sweden	51.0	13.4	1.3	1.5
	Germany	36.1	15.1	1.5	1.7
	Hungary	51.2	19.5	2.2	2.1
	Spain	52.1	13.5	3.0	2.5
Weight status category*	underweight	46.1	17.1		
	normal weight	45.6	16.5		
	overweight	43.9	16.1		
	obese	41.8	20.7		
ISCED	low	36.6	16.0		
	middle	43.9	17.0		
	high	47.1	16.2		

*25(OH)D* 25-hydroxyvitamin D, *ISCED* international standard classification of education, *UVDVC* cloud-modified vitamin D UV dose of the second last month before blood draw

*Cole and Lobstein 2012 [23].

**Fig. 2:**
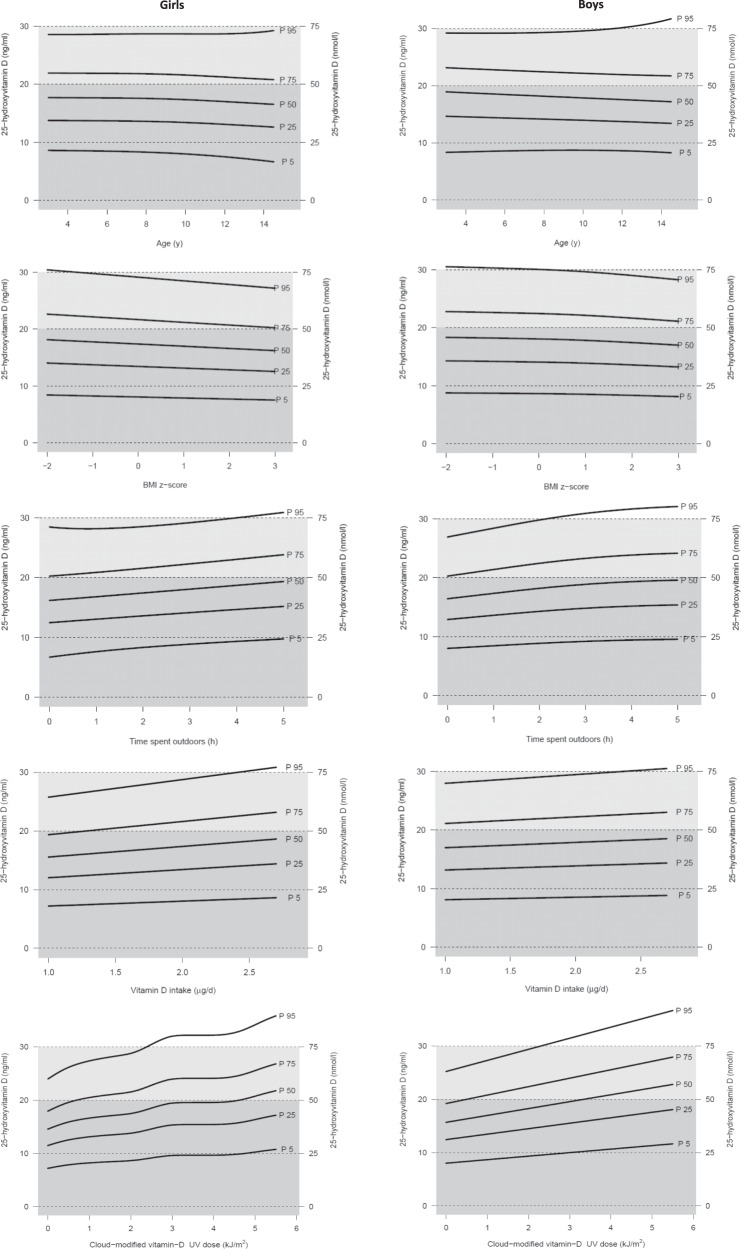
25-Hydroxy-vitamin D (25(OH)D) percentile curves for girls and boys depending on age, BMI *z* score*, time spent outdoors, dietary vitamin D intake, and UV dose (UVDVC) (separately). Gray: deficient status (<50 nmol/l), light gray: insufficient status (50-<75 nmol/l), white: sufficient status (≥75 nmol/l). *Cole and Lobstein 2012 [23]. BMI body mass index; P percentile; UVDVC, cloud-modified vitamin D UV dose of the second last month before blood draw.

Figure 2 displays the 25(OH)D percentile curves for girls and boys depending separately on age, BMI *z* score, time spent outdoors, usual dietary vitamin D intake and UVDVC of the second last month before blood draw. The respective 5th, 25th, 50th, 75th, and 95th percentiles of 25(OH)D levels are shown in Supplementary Tables 3–7. The age- and sex-specific 5th and 95th percentiles ranged from 16.5 to 73.3 nmol/l and 20.8 to 79.3 nmol/l in girls and boys, respectively (Supplementary Table 3).

The percentile curves show the steepest slope for UVDVC and time spent outdoors. Furthermore, the percentile curves depending on time spent outdoors indicate that ~50% of the children spending 5 h outdoors reached non-deficiency whereas this was true for only 25% of the children spending no time outdoors. The percentile curves depending on age are very slightly decreasing with age or approximately constant. The curves depending on usual vitamin D intake are increasing with intake which is more pronounced in girls than in boys. With increasing BMI *z* score, the curves are slightly decreasing; this is also observed considering waist-to-height-ratio but only in girls (Supplementary Fig. 1, Supplementary Table 8). The influence of BMI and time spent outdoors is evident when the curves simultaneously depending on age, UVDVC, BMI *z* score and time spent outdoors are considered: For instance, at a UVDVC of 4, >50% of the overweight 9-year-old girls were in the 25(OH)D deficient status, even if they spent 5 h outdoors, whereas >50% of normal weight girls were in the non-deficient status when spending 5 h outdoors (Supplementary Fig. 2).

### Associations of time spent outdoors and vitamin D intake with 25(OH)D

Mixed effects logistic regressions models adjusted for age, BMI *z* score, UVDVC of the second last month before blood draw, sex, country, ISCED, usual vitamin D intake, and random effect for repeated measurements show that the odds ratio (OR) for a non-deficient (i.e., sufficient or insufficient) 25(OH)D status (reference category: deficient status) by one additional hour spent outdoors was 1.21, 95% CI [1.12–1.31] (Table 4). This means that children who spent one additional hour per day outdoors had a 21% higher chance to have a non-deficient status than a deficient status. Sensitivity analyses additionally including an interaction term for UVDVC and time spent outdoors show also that the odds for a non-deficient 25(OH)D status (reference category: deficient status) were higher in children spending more time outdoors (OR = 1.20, 95% CI [1.08–1.33]). However, the interaction term did not play any role (OR = 1.01, 95% CI [0.97–1.04]). The associations are roughly the same if dietary vitamin D intake of children is additionally included in two further models showing higher odds for a non-deficient 25(OH)D status compared to a deficient status with higher usual intake: OR = 1.12, 95% CI [0.89–1.41] per µg/d for both models.

**Table 4:** Associations of time spent outdoors with serum 25-hydroxyvitamin D (nmol/l) and sensitivity analyses.

Model	Main model			Sensitivity analysis including interaction term UVDVC:time spent outdoors			Sensitivity analysis including vitamin D intake			Sensitivity analysis including vitamin D intake and interaction term UVDVC:time spent outdoors		
Sample size	3336			3336			2331			2331		
Odds ratio	OR	2.5%	97.5%	OR	2.5%	97.5%	OR	2.5%	97.5%	OR	2.5%	97.5%
**Time spent outdoors (h)**	**1.21**	**1.12**	**1.31**	**1.20**	**1.08**	**1.33**	**1.19**	**1.09**	**1.29**	**1.16**	**1.04**	**1.03**
**UVDVC:time spent outdoors**				1.01	0.97	1.04				1.01	0.97	1.06
**Vitamin D intake (µg/d)**							1.12	0.89	1.41	1.12	0.89	1.41
β estimate	β	2.5%	97.5%	β	2.5%	97.5%	β	2.5%	97.5%	β	2.5%	97.5%
**Time spent outdoors (h)**	**0.96**	**0.61**	**1.31**	**1.14**	**0.67**	**1.61**	**0.75**	**0.34**	**1.16**	**1.00**	**0.46**	**1.53**
**UVDVC:time spent outdoors**				−0.10	−0.26	0.07				−0.14	−0.34	0.06
**Vitamin D intake (µg/d)**							2.26	1.12	3.39	2.24	1.10	3.37

Model equation for linear mixed model (main model): 25(OH)D = age + BMI-*z* score + UVDVC + sex + country + ISCED + time spent outdoors + random effect for ID; analogously for mixed logistic model.

Statistically significant associations are printed in bold.

*β* β estimate, *d* day, *h* hours, *OR* odds ratio, *UVDVC* cloud-modified vitamin D UV dose of the second last month before blood draw

A positive association of 25(OH)D levels with outdoor time is also observed when continuous 25(OH)D concentrations instead of categories are considered in a linear mixed model, e.g., for the main model with an β estimate of 0.96 (95% CI [0.61–1.31]). Thus, children spending one hour more per day outdoors than other children had a 0.96 nmol/l higher 25(OH)D concentration. A sensitivity analysis including an interaction term for UVDVC and time spent outdoors shows also a positive association of 25(OH)D with time spent outdoors (β = 1.14, 95% CI [0.67–1.61]) but not at higher UVDVC (β estimate of the product term −0.10, 95% CI [−0.26–0.07]). The associations with time spent outdoors are slightly lower if usual vitamin D intake is additionally included in both aforementioned models (β = 0.75, 95% CI [0.34, 1.16]; β = 1.00, 95% CI [0.46, 1.53], respectively), showing a positive association between vitamin D intake and serum concentration of 25(OH)D (β = 2.26, 95% CI [1.12–3.39]; β = 2.24, 95% CI [1.10–3.37], respectively) (Table 4). According to the latter model, children’s serum 25(OH)D was 2.24 nmol/l higher for each additional µg vitamin D intake per day.

## Discussion

Our population-based study in European children and adolescents provides sex-specific 25(OH)D percentile curves by age, BMI *z* score, outdoor time, dietary vitamin D intake, and UVDVC based on 3606 observations of children aged 3-<15 years and evaluates the impact of modifiable determinants, particularly time spent outdoors. To our knowledge this is the first study that provides pan-European 25(OH)D reference percentiles for children and adolescents. Previous studies reported either national reference percentiles [38–40] or reference percentiles for adolescents only [10]. The percentile curves presented should not be used as reference standards for the definition of a healthy vitamin D status given that only 3% of the population had sufficient 25(OH)D levels based on the threshold of 75 nmol/l [5, 8]. However, they can help medical and biomedical personnel as well as related stakeholders and scientists to compare 25(OH)D values according to these sex-specific ranges depending on the most important determinants.

Overall, the prevalence of vitamin D deficiency (63%) or insufficiency (33%) was extremely high. Only 3% of the study population had a sufficient status. Even if serum 25(OH)D concentrations of 50-<75 nmol/l were considered as sufficient as suggested by some advisory bodies [41], e.g, the Institute of Medicine (IOM) [42], still 63% of the children in our sample would be classified as having a non-sufficient status. For the description of the 25(OH)D status of our population, we applied the recommended threshold values of the Society for Adolescent Health and Medicine as they refer to adolescents [8] although there is still no consensus on these values. Several studies in adolescents found that relatively high 25(OH)D concentrations of above 40 nmol/l [43], above 60 nmol/l [44], 75 nmol/l [45] or even 83 nmol/l [46] are needed to plateau parathyroid hormone (PTH), and 25(OH)D levels less than or equal to 40 nmol/l were associated with low forearm bone mineral density [43]. The IOM members stated as one argument against the threshold of 75 nmol/l for 25(OH)D sufficiency, that a PTH plateau at this high level was not observed in the majority of studies and that the PTH decline to a plateau depends on several factors including age, ethnicity and body composition [42]. However, one reason why a PTH plateau was not detected in most pediatric study populations could be due to the fact that the high values were rarely reached or that only a small proportion, if any, of the sample had 25(OH)D levels of >60 or 75 nmol/l. High PTH concentrations have been shown to promote the mobilization of bone minerals [47] which impairs bone mineral density [48] and may increase the risk of fractures.

In line with our results, 59% of Danish children and adolescents aged 2–17 years had serum 25(OH)D concentrations below 50 nmol/l [49]. The prevalence of 25(OH)D deficiency in teenage girls from Northern countries in winter was even 92% [50]. Because of differences in the vitamin D quantification methods applied and different distribution of values, the Vitamin D Standardization Program (VDSP) aimed at providing reliable 25(OH)D concentrations and therefore compared results from national surveys with measurements using liquid chromatography-tandem mass spectrometry (LC–tandem MS). An evaluation of the VDSP revealed that the prevalences of low serum 25(OH)D were higher after standardization. In a large cohort of German children, after standardization the prevalence of 25(OH)D < 50 nmol/l was 30.7% in summer (Apr–Oct) and 64.3% in winter (Nov–March) [51, 52].

The risk of vitamin D deficiency increased if children lived in a place at high latitude with resulting low UVDVC, spent little time outdoors, had a low vitamin D intake, had overweight or obesity, and were older. Also female sex and a low educational attainment of the parents seem to be associated with a lower 25(OH)D status. A recent German study reported percentiles of 25(OH)D concentrations for summer and winter. In summer, the 50th percentile of the comparable age group with our study (3–14 years) ranged from 72.75 to 55.25 nmol/l in boys and 72.78 to 52.08 nmol/l in girls. In winter, 25(OH)D concentrations were considerably lower and ranged from 54.98 to 43.53 nmol/l in boys and from 53.35 to 41.03 nmol/l in girls [39]. We observed a similar age-dependent decrease but mean 25(OH)D concentrations in Northern countries except for Sweden were even lower in our cohort. Considering the high variability of 25(OH)D status over the years by latitude, by the respective UV radiation and the cloud cover, our study provides accurate reference percentiles by UVDVC instead of only considering season. As reported from cohorts of adults [53], children and adolescents [54, 55], we have also observed a high degree of tracking of 25(OH)D values over six years (*r* = 0.47); around two thirds of the children remained in the same 25(OH)D status category.

Among the modifiable determinants of vitamin D status in our study, time spent outdoors had the strongest effect. In the rough model, children who spent one additional hour per day outdoors had a 21% higher chance to have a non-deficient status. Because it can be assumed that this chance is influenced by the UVDVC, we included an interaction term UVDVC * time spent outdoors which resulted in negligible changes. This is possibly due to the fact that UVDVC alone has a very large effect on 25(OH)D (e.g., OR = 1.6, 95% CI [1.44, 1.78], in the sensitivity analysis including interaction term UVDVC:time spent outdoors).

Although percentile curves indicated that UVDVC had the strongest effect on 25(OH)D, the latitude as the strongest influencing factor of UV radiation of a region was still not a reliable predictor of 25(OH)D. Italian children had the third-highest average UVDVC among our study locations, but Italian children (together with those from Germany and Estonia) had the lowest mean 25(OH)D level. In contrast, the mean 25(OH)D of Swedish children who lived in the area with the third-lowest UVDVC was above 50 nmol/l and thus in the same range as in children from Hungary or Spain. This could be partly due to the Swedish vitamin D fortification policy which covered the mandatory fortification of some dairy products at that time. Nevertheless, the Swedish national survey revealed that in adults only 12% of the dietary vitamin D intake originated from milk products [56] but children usually drink more milk. In addition, a high intake of fish [57], less TV and media time and more time spent physically active [58] indicating more time spent outdoors as well as a lower prevalence of overweight/obesity [59] as reported for Swedish children may have contributed to their better 25(OH)D status. A poor vitamin D status in Southern Italy has been shown previously and was explained by low sun exposure on a more pigmented skin and little access to vitamin D rich food like oily fish or cod liver oil [41]. Accordingly, previous studies in the IDEFICS population have revealed that children from Italy had the lowest compliance to physical activity recommendations [60] which may indicate that they spent little time outdoors. In addition, they had the highest prevalence of overweight and obesity (42.4%) [59] which was inversely associated with 25(OH)D in previous studies [39, 61].

Mean usual vitamin D intake in our study was much lower than recommended by EFSA and other advisory bodies who suggest an intake between 5 and 15 µg/d for children [7, 62]. Although serum 25(OH)D mirrors both, dietary vitamin D intake and contributions from dermal production in response to sunlight exposure, we observed a positive association of usual intake and serum 25(OH)D. Children with an intake of 1 µg vitamin D more than other children had an 2.26 nmol/l higher serum 25(OH)D, which was within the range of 1.42–2.90 nmol/l and 0.24–3.19 nmol/l previously reported for children who received vitamin D supplements and fortified foods, respectively [63]. However, a recent meta-analysis reported an increment of 1.6 nmol/l per 2.5 µg/d from low-dose supplementation which corresponds to an increment of only 0.64 nmol/l per each additional 1 µg/d [64].

In our regression analysis, the inverse association between BMI and 25(OH)D was negligibly/very small (data not shown) which suggest that other determinants included in the model like UVDVC and time spent outdoors were more crucial determinants.

### Limitations and strengths

Although our study demonstrates the role of numerous determinants for the 25(OH)D level, several influencing factors could not be considered. This includes the contribution of vitamin D from fortified foods except for those considered in the nutrient data base, the skin pigmentation of participants as well as the area of skin exposed to the sun and the use of sunscreen. In addition, we have used the average UVDVC of the second last month before blood draw which means that the lag time can vary depending on the date of blood draw which may have slightly influenced our results. As the UVDVC was not available for the study center locations except for Tallinn and Nicosia, we have taken the values from cities in the same country which were somewhat distant from the study centers. Therefore, the latitude between these locations slightly varied and also the cloud cover may have differed. Also, estimates of usual vitamin D intake from repeated 24-hour dietary recalls are subject to uncertainty, which we tried to reduce using the NCI method considering the daily variation in diet.

On the other hand our study has several strengths as it provides percentile curves of serum 25(OH)D concentrations calculated by GAMLSS based on a large population of children and adolescents from Northern, Eastern, Western and Southern regions across Europe. Participants of this cohort study were extensively phenotyped in terms of lifestyle behaviors including dietary intake, anthropometric measurements and biomarker data. The design of our study allowed association analysis considering several determinants of 25(OH)D concentrations at baseline and after six years of follow-up. Serum 25(OH)D levels were analysed in a central laboratory. All examinations and measurements were conducted based on highly standardized protocols. In addition, adherence to standard operating procedures was ensured for field personnel by a central training and by site visits [20, 21].

## Conclusion

Our study revealed that a very high proportion of European children and adolescents had insufficient vitamin D status. As UVDVC and outdoor time were the most important determinants of vitamin D status, measures to expand the time spent outdoors by promoting outdoor activities in settings like kindergartens and schools should be implemented. This would also help to prevent overweight and obesity which additionally affect serum 25(OH)D concentrations, although potential adverse effects of sunlight exposure like skin cancer need to be taken into account during outdoor time. In addition, if not yet in place, systematic mandatory fortification of foods like dairy products and bread or vitamin D supplementation should be considered to increase vitamin D intake as previously suggested [7, 41, 65]. These measures will help to counteract vitamin D deficiency in young European populations. Measurement of serum 25(OH)D levels should be implemented in pediatric routine to guide targeted supplementation [66], particularly in children with increased risk of deficiency, e.g., children with low outdoor time, insufficient UV exposure, obesity, low or no consumption of oily fish, a more pigmented skin or low parental educational attainment.

## Supplementary information

Supplementary Material
