# Supplementary material for: 25-Hydroxyvitamin D reference percentiles and the role of their determinants among European children and adolescents
Source: Eur J Clin Nutr. 2021 Jul 23;76(4):564–73. doi: 10.1038/s41430-021-00985-4 (PMC8993686; doi:10.1038/s41430-021-00985-4)
Supplement: Supplementary file 1 — Supplementary Material [file 41430_2021_985_MOESM1_ESM.docx]

**Supplementary Material**

**25-Hydroxyvitamin D reference percentiles and the role of their determinants among European children and adolescents**

**Running title: 25(OH)D percentiles and their determinants among European children**

Maike Wolters^1*#^ , Timm Intemann^1*^, Paola Russo^2^, Luis A Moreno^3^, Dénes Molnár^4^, Toomas Veidebaum^5^, Michael Tornaritis^6^, Stefaan De Henauw^7^, Gabriele Eiben^8,9^, Wolfgang Ahrens^1,10^, Anna Floegel^1^ on behalf of the IDEFICS and I.Family consortia

^1^Leibniz Institute for Prevention Research and Epidemiology - BIPS, 28359 Bremen, Germany

^2^Institute of Food Sciences, National Research Council, 83100 Avellino, Italy

^3^GENUD (Growth, Exercise, Nutrition and Development) Research Group, Faculty of Health Sciences, Universidad de Zaragoza, Instituto Agroalimentario de Aragón (IA2), Instituto de Investigación Sanitaria Aragón (IIS Aragón), 50009 Zaragoza, Spain and Centro de Investigación Biomédica en Red de Fisiopatología de la Obesidad y Nutrición (CIBERObn), Instituto de Salud Carlos III, 28029 Madrid, Spain

^4^Department of Pediatrics, Medical School, University of Pécs, 7623 Pécs, Hungary

^5^National Institute for Health Development, Estonian Centre of Behavioral and Health Sciences, 11619 Tallinn, Estonia

^6^Research and Education Institute of Child Health, 2035 Strovolos, Cyprus

^7^Department of Public Health and Primary Care, Ghent University, 9000 Ghent, Belgium

^8^Department of Public Health and Community Medicine, University of Gothenburg, 405 30 Gothenburg, Sweden

^9^Department of Public Health, School of Health Sciences, University of Skövde, Skövde, Sweden

^10^Institute of Statistics, Faculty of Mathematics and Computer Science, University of Bremen, 28359 Bremen, Germany

*These authors contributed equally to this work.

^#^**Corresponding author**

Dr. Maike Wolters

Leibniz Institute for Prevention Research and Epidemiology – BIPS

Achterstr. 30

28359 Bremen, Germany

Email: [wolters@leibniz-bips.de](mailto:wolters@leibniz-bips.de); [sec-epi@leibniz-bips.de](mailto:wolters@leibniz-bips.de)

Phone: +49 421 218-56845

Fax: +49 421 218-56821

**Supplementary Table 1: Final selected GAMLSS models for percentile curve estimation**

**Supplementary Table 2: Number of children by vitamin D status category at T0 and T3**

| **25-Hydroxy-Vitamin D levels** |  | **T3** |  |  |
| --- | --- | --- | --- | --- |
| **T0** | **<50 nmol/l** | **50-75 nmol/l** | **>=75 nmol/l** | **Sum** |
| **<50 nmol/l** | 652 | 213 | 19 | 884 |
| **50-75 nmol/l** | 214 | 256 | 42 | 512 |
| **>=75 nmol/l** | 4 | 29 | 6 | 39 |
| **Sum** | 870 | 498 | 67 | 1435 |

**Supplementary Table 3: Sex- and age-specific percentiles (P) of 25-hydroxyvitamin D (nmol/l) in girls and boys**

| **Percentiles for girls** | **Percentiles for boys** |
| --- | --- |
| \| **Age (y)** \| **P5** \| **P25** \| **P50** \| **P75** \| **P95** \| \| --- \| --- \| --- \| --- \| --- \| --- \| | \| **Age (y)** \| **P5** \| **P25** \| **P50** \| **P75** \| **P95** \| \| --- \| --- \| --- \| --- \| --- \| --- \| |
| \| 3.0 \| 21.5 \| 34.5 \| 44.3 \| 54.8 \| 71.5 \| \| --- \| --- \| --- \| --- \| --- \| --- \| \| 3.1 \| 21.5 \| 34.5 \| 44.3 \| 54.8 \| 71.5 \| \| 3.2 \| 21.5 \| 34.5 \| 44.3 \| 54.8 \| 71.5 \| \| 3.3 \| 21.5 \| 34.5 \| 44.3 \| 54.8 \| 71.5 \| \| 3.4 \| 21.5 \| 34.5 \| 44.3 \| 54.8 \| 71.5 \| \| 3.5 \| 21.5 \| 34.5 \| 44.3 \| 54.8 \| 71.5 \| \| 3.6 \| 21.5 \| 34.3 \| 44.3 \| 54.8 \| 71.5 \| \| 3.7 \| 21.5 \| 34.3 \| 44.3 \| 54.8 \| 71.5 \| \| 3.8 \| 21.5 \| 34.3 \| 44.3 \| 54.8 \| 71.5 \| \| 3.9 \| 21.5 \| 34.3 \| 44.3 \| 54.8 \| 71.5 \| \| 4.0 \| 21.5 \| 34.3 \| 44.3 \| 54.8 \| 71.5 \| \| 4.1 \| 21.5 \| 34.3 \| 44.3 \| 54.8 \| 71.5 \| \| 4.2 \| 21.5 \| 34.3 \| 44.3 \| 54.8 \| 71.5 \| \| 4.3 \| 21.3 \| 34.3 \| 44.3 \| 54.8 \| 71.5 \| \| 4.4 \| 21.3 \| 34.3 \| 44.3 \| 54.8 \| 71.5 \| \| 4.5 \| 21.3 \| 34.3 \| 44.3 \| 54.8 \| 71.5 \| \| 4.6 \| 21.3 \| 34.3 \| 44.3 \| 54.8 \| 71.5 \| \| 4.7 \| 21.3 \| 34.3 \| 44.3 \| 54.8 \| 71.5 \| \| 4.8 \| 21.3 \| 34.3 \| 44.3 \| 54.8 \| 71.5 \| \| 4.9 \| 21.3 \| 34.3 \| 44.3 \| 54.8 \| 71.5 \| \| 5.0 \| 21.3 \| 34.3 \| 44.3 \| 54.8 \| 71.5 \| \| 5.1 \| 21.3 \| 34.3 \| 44.3 \| 54.8 \| 71.5 \| \| 5.2 \| 21.3 \| 34.3 \| 44.3 \| 54.8 \| 71.5 \| \| 5.3 \| 21.3 \| 34.3 \| 44.3 \| 54.8 \| 71.5 \| \| 5.4 \| 21.3 \| 34.3 \| 44.3 \| 54.8 \| 71.5 \| \| 5.5 \| 21.3 \| 34.3 \| 44.3 \| 54.8 \| 71.5 \| \| 5.6 \| 21.3 \| 34.3 \| 44.3 \| 54.8 \| 71.5 \| \| **Percentiles for girls** \| \| \| \| \| \| \| **Age (y)** \| **P5** \| **P25** \| **P50** \| **P75** \| **P95** \| \| 5.7 \| 21.3 \| 34.3 \| 44.3 \| 54.8 \| 71.5 \| \| 5.8 \| 21.3 \| 34.3 \| 44.0 \| 54.8 \| 71.5 \| \| 5.9 \| 21.3 \| 34.3 \| 44.0 \| 54.8 \| 71.5 \| \| 6.0 \| 21.3 \| 34.3 \| 44.0 \| 54.8 \| 71.5 \| \| 6.1 \| 21.3 \| 34.3 \| 44.0 \| 54.8 \| 71.8 \| \| 6.2 \| 21.0 \| 34.3 \| 44.0 \| 54.8 \| 71.8 \| \| 6.3 \| 21.0 \| 34.3 \| 44.0 \| 54.8 \| 71.8 \| \| 6.4 \| 21.0 \| 34.3 \| 44.0 \| 54.8 \| 71.8 \| \| 6.5 \| 21.0 \| 34.3 \| 44.0 \| 54.8 \| 71.8 \| \| 6.6 \| 21.0 \| 34.3 \| 44.0 \| 54.8 \| 71.8 \| \| 6.7 \| 21.0 \| 34.3 \| 44.0 \| 54.8 \| 71.8 \| \| 6.8 \| 21.0 \| 34.3 \| 44.0 \| 54.8 \| 71.8 \| \| 6.9 \| 21.0 \| 34.3 \| 44.0 \| 54.8 \| 71.8 \| \| 7.0 \| 21.0 \| 34.3 \| 44.0 \| 54.8 \| 71.8 \| \| 7.1 \| 21.0 \| 34.3 \| 44.0 \| 54.8 \| 71.8 \| \| 7.2 \| 21.0 \| 34.3 \| 44.0 \| 54.8 \| 71.8 \| \| 7.3 \| 21.0 \| 34.0 \| 44.0 \| 54.5 \| 71.8 \| \| 7.4 \| 21.0 \| 34.0 \| 44.0 \| 54.5 \| 71.8 \| \| 7.5 \| 20.8 \| 34.0 \| 44.0 \| 54.5 \| 71.8 \| \| 7.6 \| 20.8 \| 34.0 \| 44.0 \| 54.5 \| 71.8 \| \| 7.7 \| 20.8 \| 34.0 \| 44.0 \| 54.5 \| 71.8 \| \| 7.8 \| 20.8 \| 34.0 \| 44.0 \| 54.5 \| 71.8 \| \| 7.9 \| 20.8 \| 34.0 \| 44.0 \| 54.5 \| 71.8 \| \| 8.0 \| 20.8 \| 34.0 \| 44.0 \| 54.5 \| 71.8 \| \| 8.1 \| 20.8 \| 34.0 \| 44.0 \| 54.5 \| 71.8 \| \| 8.2 \| 20.8 \| 34.0 \| 43.8 \| 54.5 \| 71.8 \| \| 8.3 \| 20.8 \| 34.0 \| 43.8 \| 54.5 \| 71.8 \| \| 8.4 \| 20.5 \| 34.0 \| 43.8 \| 54.5 \| 71.8 \| \| 8.5 \| 20.5 \| 34.0 \| 43.8 \| 54.5 \| 71.8 \| \| **Percentiles for girls** \| \| \| \| \| \| \| **Age (y)** \| **P5** \| **P25** \| **P50** \| **P75** \| **P95** \| \| 8.6 \| 20.5 \| 34.0 \| 43.8 \| 54.5 \| 71.8 \| \| 8.7 \| 20.5 \| 34.0 \| 43.8 \| 54.5 \| 71.8 \| \| 8.8 \| 20.5 \| 33.8 \| 43.8 \| 54.3 \| 71.8 \| \| 8.9 \| 20.5 \| 33.8 \| 43.8 \| 54.3 \| 71.8 \| \| 9.0 \| 20.5 \| 33.8 \| 43.8 \| 54.3 \| 71.8 \| \| 9.1 \| 20.3 \| 33.8 \| 43.8 \| 54.3 \| 71.8 \| \| 9.2 \| 20.3 \| 33.8 \| 43.8 \| 54.3 \| 71.8 \| \| 9.3 \| 20.3 \| 33.8 \| 43.5 \| 54.3 \| 71.8 \| \| 9.4 \| 20.3 \| 33.8 \| 43.5 \| 54.3 \| 71.8 \| \| 9.5 \| 20.3 \| 33.8 \| 43.5 \| 54.3 \| 71.8 \| \| 9.6 \| 20.3 \| 33.8 \| 43.5 \| 54.3 \| 71.8 \| \| 9.7 \| 20.0 \| 33.5 \| 43.5 \| 54.0 \| 71.8 \| \| 9.8 \| 20.0 \| 33.5 \| 43.5 \| 54.0 \| 71.8 \| \| 9.9 \| 20.0 \| 33.5 \| 43.5 \| 54.0 \| 71.8 \| \| 10.0 \| 20.0 \| 33.5 \| 43.5 \| 54.0 \| 71.8 \| \| 10.1 \| 20.0 \| 33.5 \| 43.3 \| 54.0 \| 71.8 \| \| 10.2 \| 19.8 \| 33.5 \| 43.3 \| 54.0 \| 71.8 \| \| 10.3 \| 19.8 \| 33.5 \| 43.3 \| 54.0 \| 71.8 \| \| 10.4 \| 19.8 \| 33.5 \| 43.3 \| 53.8 \| 71.8 \| \| 10.5 \| 19.8 \| 33.3 \| 43.3 \| 53.8 \| 71.8 \| \| 10.6 \| 19.8 \| 33.3 \| 43.3 \| 53.8 \| 71.8 \| \| 10.7 \| 19.5 \| 33.3 \| 43.0 \| 53.8 \| 71.8 \| \| 10.8 \| 19.5 \| 33.3 \| 43.0 \| 53.8 \| 71.8 \| \| 10.9 \| 19.5 \| 33.3 \| 43.0 \| 53.8 \| 71.8 \| \| 11.0 \| 19.5 \| 33.3 \| 43.0 \| 53.5 \| 71.8 \| \| 11.1 \| 19.3 \| 33.0 \| 43.0 \| 53.5 \| 71.8 \| \| 11.2 \| 19.3 \| 33.0 \| 43.0 \| 53.5 \| 71.8 \| \| 11.3 \| 19.3 \| 33.0 \| 42.8 \| 53.5 \| 71.8 \| \| 11.4 \| 19.3 \| 33.0 \| 42.8 \| 53.5 \| 71.8 \| \| **Percentiles for girls** \| \| \| \| \| \| \| **Age (y)** \| **P5** \| **P25** \| **P50** \| **P75** \| **P95** \| \| 11.5 \| 19.0 \| 33.0 \| 42.8 \| 53.3 \| 71.8 \| \| 11.6 \| 19.0 \| 33.0 \| 42.8 \| 53.3 \| 71.8 \| \| 11.7 \| 19.0 \| 32.8 \| 42.8 \| 53.3 \| 71.8 \| \| 11.8 \| 18.8 \| 32.8 \| 42.8 \| 53.3 \| 71.8 \| \| 11.9 \| 18.8 \| 32.8 \| 42.5 \| 53.3 \| 71.8 \| \| 12.0 \| 18.8 \| 32.8 \| 42.5 \| 53.0 \| 71.8 \| \| 12.1 \| 18.8 \| 32.8 \| 42.5 \| 53.0 \| 71.8 \| \| 12.2 \| 18.5 \| 32.8 \| 42.5 \| 53.0 \| 71.8 \| \| 12.3 \| 18.5 \| 32.5 \| 42.5 \| 53.0 \| 71.8 \| \| 12.4 \| 18.5 \| 32.5 \| 42.3 \| 53.0 \| 71.8 \| \| 12.5 \| 18.3 \| 32.5 \| 42.3 \| 53.0 \| 71.8 \| \| 12.6 \| 18.3 \| 32.5 \| 42.3 \| 52.8 \| 72.0 \| \| 12.7 \| 18.3 \| 32.5 \| 42.3 \| 52.8 \| 72.0 \| \| 12.8 \| 18.0 \| 32.3 \| 42.3 \| 52.8 \| 72.0 \| \| 12.9 \| 18.0 \| 32.3 \| 42.0 \| 52.8 \| 72.0 \| \| 13.0 \| 18.0 \| 32.3 \| 42.0 \| 52.8 \| 72.0 \| \| 13.1 \| 17.8 \| 32.3 \| 42.0 \| 52.5 \| 72.0 \| \| 13.2 \| 17.8 \| 32.3 \| 42.0 \| 52.5 \| 72.0 \| \| 13.3 \| 17.8 \| 32.0 \| 42.0 \| 52.5 \| 72.3 \| \| 13.4 \| 17.5 \| 32.0 \| 42.0 \| 52.5 \| 72.3 \| \| 13.5 \| 17.5 \| 32.0 \| 41.8 \| 52.5 \| 72.3 \| \| 13.6 \| 17.5 \| 32.0 \| 41.8 \| 52.5 \| 72.3 \| \| 13.7 \| 17.3 \| 32.0 \| 41.8 \| 52.3 \| 72.5 \| \| 13.8 \| 17.3 \| 32.0 \| 41.8 \| 52.3 \| 72.5 \| \| 13.9 \| 17.3 \| 31.8 \| 41.8 \| 52.3 \| 72.5 \| \| 14.0 \| 17.0 \| 31.8 \| 41.5 \| 52.3 \| 72.8 \| \| 14.1 \| 17.0 \| 31.8 \| 41.5 \| 52.3 \| 72.8 \| \| 14.2 \| 16.8 \| 31.8 \| 41.5 \| 52.3 \| 72.8 \| \| 14.3 \| 16.8 \| 31.8 \| 41.5 \| 52.0 \| 73.0 \| \| **Percentiles for girls** \| \| \| \| \| \| \| **Age (y)** \| **P5** \| **P25** \| **P50** \| **P75** \| **P95** \| \| 14.4 \| 16.8 \| 31.5 \| 41.5 \| 52.0 \| 73.0 \| \| 14.5 \| 16.5 \| 31.5 \| 41.3 \| 52.0 \| 73.3 \| | \| 3.0 \| 20.8 \| 36.8 \| 47.3 \| 57.8 \| 73.0 \| \| --- \| --- \| --- \| --- \| --- \| --- \| \| 3.1 \| 21.0 \| 36.5 \| 47.3 \| 57.8 \| 73.0 \| \| 3.2 \| 21.0 \| 36.5 \| 47.3 \| 57.8 \| 73.0 \| \| 3.3 \| 21.0 \| 36.5 \| 47.3 \| 57.8 \| 73.0 \| \| 3.4 \| 21.0 \| 36.5 \| 47.3 \| 57.8 \| 73.0 \| \| 3.5 \| 21.0 \| 36.5 \| 47.3 \| 57.8 \| 73.0 \| \| 3.6 \| 21.0 \| 36.5 \| 47.0 \| 57.8 \| 73.0 \| \| 3.7 \| 21.0 \| 36.5 \| 47.0 \| 57.5 \| 73.0 \| \| 3.8 \| 21.0 \| 36.5 \| 47.0 \| 57.5 \| 73.0 \| \| 3.9 \| 21.0 \| 36.5 \| 47.0 \| 57.5 \| 73.0 \| \| 4.0 \| 21.0 \| 36.5 \| 47.0 \| 57.5 \| 73.0 \| \| 4.1 \| 21.0 \| 36.3 \| 47.0 \| 57.5 \| 73.0 \| \| 4.2 \| 21.0 \| 36.3 \| 46.8 \| 57.5 \| 73.0 \| \| 4.3 \| 21.0 \| 36.3 \| 46.8 \| 57.5 \| 73.0 \| \| 4.4 \| 21.3 \| 36.3 \| 46.8 \| 57.3 \| 73.0 \| \| 4.5 \| 21.3 \| 36.3 \| 46.8 \| 57.3 \| 73.0 \| \| 4.6 \| 21.3 \| 36.3 \| 46.8 \| 57.3 \| 73.0 \| \| 4.7 \| 21.3 \| 36.3 \| 46.8 \| 57.3 \| 73.0 \| \| 4.8 \| 21.3 \| 36.3 \| 46.5 \| 57.3 \| 73.0 \| \| 4.9 \| 21.3 \| 36.3 \| 46.5 \| 57.3 \| 73.0 \| \| 5.0 \| 21.3 \| 36.3 \| 46.5 \| 57.3 \| 73.0 \| \| 5.1 \| 21.3 \| 36.0 \| 46.5 \| 57.0 \| 73.0 \| \| 5.2 \| 21.3 \| 36.0 \| 46.5 \| 57.0 \| 73.0 \| \| 5.3 \| 21.3 \| 36.0 \| 46.5 \| 57.0 \| 73.0 \| \| 5.4 \| 21.3 \| 36.0 \| 46.5 \| 57.0 \| 73.0 \| \| 5.5 \| 21.3 \| 36.0 \| 46.3 \| 57.0 \| 73.0 \| \| 5.6 \| 21.5 \| 36.0 \| 46.3 \| 57.0 \| 73.0 \| \| **Percentiles for boys** \| \| \| \| \| \| \| **Age (y)** \| **P5** \| **P25** \| **P50** \| **P75** \| **P95** \| \| 5.7 \| 21.5 \| 36.0 \| 46.3 \| 57.0 \| 73.0 \| \| 5.8 \| 21.5 \| 36.0 \| 46.3 \| 56.8 \| 73.0 \| \| 5.9 \| 21.5 \| 36.0 \| 46.3 \| 56.8 \| 73.0 \| \| 6.0 \| 21.5 \| 36.0 \| 46.3 \| 56.8 \| 73.0 \| \| 6.1 \| 21.5 \| 35.8 \| 46.3 \| 56.8 \| 73.0 \| \| 6.2 \| 21.5 \| 35.8 \| 46.0 \| 56.8 \| 73.0 \| \| 6.3 \| 21.5 \| 35.8 \| 46.0 \| 56.8 \| 73.0 \| \| 6.4 \| 21.5 \| 35.8 \| 46.0 \| 56.8 \| 73.0 \| \| 6.5 \| 21.5 \| 35.8 \| 46.0 \| 56.5 \| 73.0 \| \| 6.6 \| 21.5 \| 35.8 \| 46.0 \| 56.5 \| 73.0 \| \| 6.7 \| 21.5 \| 35.8 \| 46.0 \| 56.5 \| 73.0 \| \| 6.8 \| 21.5 \| 35.8 \| 45.8 \| 56.5 \| 73.0 \| \| 6.9 \| 21.5 \| 35.8 \| 45.8 \| 56.5 \| 73.0 \| \| 7.0 \| 21.5 \| 35.8 \| 45.8 \| 56.5 \| 73.0 \| \| 7.1 \| 21.5 \| 35.5 \| 45.8 \| 56.5 \| 73.3 \| \| 7.2 \| 21.8 \| 35.5 \| 45.8 \| 56.3 \| 73.3 \| \| 7.3 \| 21.8 \| 35.5 \| 45.8 \| 56.3 \| 73.3 \| \| 7.4 \| 21.8 \| 35.5 \| 45.8 \| 56.3 \| 73.3 \| \| 7.5 \| 21.8 \| 35.5 \| 45.5 \| 56.3 \| 73.3 \| \| 7.6 \| 21.8 \| 35.5 \| 45.5 \| 56.3 \| 73.3 \| \| 7.7 \| 21.8 \| 35.5 \| 45.5 \| 56.3 \| 73.3 \| \| 7.8 \| 21.8 \| 35.5 \| 45.5 \| 56.3 \| 73.3 \| \| 7.9 \| 21.8 \| 35.5 \| 45.5 \| 56.0 \| 73.3 \| \| 8.0 \| 21.8 \| 35.5 \| 45.5 \| 56.0 \| 73.3 \| \| 8.1 \| 21.8 \| 35.3 \| 45.5 \| 56.0 \| 73.3 \| \| 8.2 \| 21.8 \| 35.3 \| 45.3 \| 56.0 \| 73.3 \| \| 8.3 \| 21.8 \| 35.3 \| 45.3 \| 56.0 \| 73.3 \| \| 8.4 \| 21.8 \| 35.3 \| 45.3 \| 56.0 \| 73.5 \| \| 8.5 \| 21.8 \| 35.3 \| 45.3 \| 56.0 \| 73.5 \| \| **Percentiles for boys** \| \| \| \| \| \| \| **Age (y)** \| **P5** \| **P25** \| **P50** \| **P75** \| **P95** \| \| 8.6 \| 21.8 \| 35.3 \| 45.3 \| 56.0 \| 73.5 \| \| 8.7 \| 21.8 \| 35.3 \| 45.3 \| 55.8 \| 73.5 \| \| 8.8 \| 21.8 \| 35.3 \| 45.0 \| 55.8 \| 73.5 \| \| 8.9 \| 21.8 \| 35.3 \| 45.0 \| 55.8 \| 73.5 \| \| 9.0 \| 21.8 \| 35.3 \| 45.0 \| 55.8 \| 73.5 \| \| 9.1 \| 21.8 \| 35.0 \| 45.0 \| 55.8 \| 73.5 \| \| 9.2 \| 21.8 \| 35.0 \| 45.0 \| 55.8 \| 73.8 \| \| 9.3 \| 21.8 \| 35.0 \| 45.0 \| 55.8 \| 73.8 \| \| 9.4 \| 21.8 \| 35.0 \| 45.0 \| 55.8 \| 73.8 \| \| 9.5 \| 21.8 \| 35.0 \| 44.8 \| 55.5 \| 73.8 \| \| 9.6 \| 21.8 \| 35.0 \| 44.8 \| 55.5 \| 73.8 \| \| 9.7 \| 21.8 \| 35.0 \| 44.8 \| 55.5 \| 73.8 \| \| 9.8 \| 21.8 \| 35.0 \| 44.8 \| 55.5 \| 74.0 \| \| 9.9 \| 21.8 \| 35.0 \| 44.8 \| 55.5 \| 74.0 \| \| 10.0 \| 21.8 \| 35.0 \| 44.8 \| 55.5 \| 74.0 \| \| 10.1 \| 21.8 \| 34.8 \| 44.8 \| 55.5 \| 74.0 \| \| 10.2 \| 21.8 \| 34.8 \| 44.5 \| 55.5 \| 74.0 \| \| 10.3 \| 21.8 \| 34.8 \| 44.5 \| 55.3 \| 74.3 \| \| 10.4 \| 21.8 \| 34.8 \| 44.5 \| 55.3 \| 74.3 \| \| 10.5 \| 21.8 \| 34.8 \| 44.5 \| 55.3 \| 74.3 \| \| 10.6 \| 21.8 \| 34.8 \| 44.5 \| 55.3 \| 74.3 \| \| 10.7 \| 21.8 \| 34.8 \| 44.5 \| 55.3 \| 74.3 \| \| 10.8 \| 21.8 \| 34.8 \| 44.3 \| 55.3 \| 74.5 \| \| 10.9 \| 21.8 \| 34.8 \| 44.3 \| 55.3 \| 74.5 \| \| 11.0 \| 21.8 \| 34.5 \| 44.3 \| 55.3 \| 74.5 \| \| 11.1 \| 21.8 \| 34.5 \| 44.3 \| 55.0 \| 74.8 \| \| 11.2 \| 21.8 \| 34.5 \| 44.3 \| 55.0 \| 74.8 \| \| 11.3 \| 21.8 \| 34.5 \| 44.3 \| 55.0 \| 74.8 \| \| 11.4 \| 21.8 \| 34.5 \| 44.3 \| 55.0 \| 74.8 \| \| **Percentiles for boys** \| \| \| \| \| \| \| **Age (y)** \| **P5** \| **P25** \| **P50** \| **P75** \| **P95** \| \| 11.5 \| 21.8 \| 34.5 \| 44.0 \| 55.0 \| 75.0 \| \| 11.6 \| 21.5 \| 34.5 \| 44.0 \| 55.0 \| 75.0 \| \| 11.7 \| 21.5 \| 34.5 \| 44.0 \| 55.0 \| 75.0 \| \| 11.8 \| 21.5 \| 34.5 \| 44.0 \| 55.0 \| 75.3 \| \| 11.9 \| 21.5 \| 34.3 \| 44.0 \| 55.0 \| 75.3 \| \| 12.0 \| 21.5 \| 34.3 \| 44.0 \| 54.8 \| 75.5 \| \| 12.1 \| 21.5 \| 34.3 \| 44.0 \| 54.8 \| 75.5 \| \| 12.2 \| 21.5 \| 34.3 \| 43.8 \| 54.8 \| 75.5 \| \| 12.3 \| 21.5 \| 34.3 \| 43.8 \| 54.8 \| 75.8 \| \| 12.4 \| 21.5 \| 34.3 \| 43.8 \| 54.8 \| 75.8 \| \| 12.5 \| 21.5 \| 34.3 \| 43.8 \| 54.8 \| 76.0 \| \| 12.6 \| 21.5 \| 34.3 \| 43.8 \| 54.8 \| 76.0 \| \| 12.7 \| 21.5 \| 34.0 \| 43.8 \| 54.8 \| 76.3 \| \| 12.8 \| 21.3 \| 34.0 \| 43.8 \| 54.8 \| 76.3 \| \| 12.9 \| 21.3 \| 34.0 \| 43.5 \| 54.8 \| 76.5 \| \| 13.0 \| 21.3 \| 34.0 \| 43.5 \| 54.5 \| 76.5 \| \| 13.1 \| 21.3 \| 34.0 \| 43.5 \| 54.5 \| 76.8 \| \| 13.2 \| 21.3 \| 34.0 \| 43.5 \| 54.5 \| 76.8 \| \| 13.3 \| 21.3 \| 34.0 \| 43.5 \| 54.5 \| 77.0 \| \| 13.4 \| 21.3 \| 34.0 \| 43.5 \| 54.5 \| 77.3 \| \| 13.5 \| 21.0 \| 33.8 \| 43.3 \| 54.5 \| 77.3 \| \| 13.6 \| 21.0 \| 33.8 \| 43.3 \| 54.5 \| 77.5 \| \| 13.7 \| 21.0 \| 33.8 \| 43.3 \| 54.5 \| 77.8 \| \| 13.8 \| 21.0 \| 33.8 \| 43.3 \| 54.5 \| 77.8 \| \| 13.9 \| 21.0 \| 33.8 \| 43.3 \| 54.5 \| 78.0 \| \| 14.0 \| 21.0 \| 33.8 \| 43.3 \| 54.5 \| 78.3 \| \| 14.1 \| 20.8 \| 33.8 \| 43.3 \| 54.5 \| 78.5 \| \| 14.2 \| 20.8 \| 33.8 \| 43.0 \| 54.3 \| 78.5 \| \| 14.3 \| 20.8 \| 33.5 \| 43.0 \| 54.3 \| 78.8 \| \| **Percentiles for boys** \| \| \| \| \| \| \| **Age (y)** \| **P5** \| **P25** \| **P50** \| **P75** \| **P95** \| \| 14.4 \| 20.8 \| 33.5 \| 43.0 \| 54.3 \| 79.0 \| \| 14.5 \| 20.8 \| 33.5 \| 43.0 \| 54.3 \| 79.3 \| |

**Supplementary Table 4: Sex-specific percentiles (P) of 25-hydroxyvitamin D (nmol/l) in girls and boys depending on BMI z-score***

| **Percentiles for girls** | **Percentiles for boys** |
| --- | --- |
| \| **BMI z-score** \| **P5** \| **P25** \| **P50** \| **P75** \| **P95** \| \| --- \| --- \| --- \| --- \| --- \| --- \| | \| **BMI z-score** \| **P5** \| **P25** \| **P50** \| **P75** \| **P95** \| \| --- \| --- \| --- \| --- \| --- \| --- \| |
| \| -2.0 \| 21.0 \| 35.0 \| 45.3 \| 56.5 \| 76.3 \| \| --- \| --- \| --- \| --- \| --- \| --- \| \| -1.9 \| 21.0 \| 35.0 \| 45.3 \| 56.5 \| 76.0 \| \| -1.8 \| 21.0 \| 35.0 \| 45.3 \| 56.5 \| 75.8 \| \| -1.7 \| 20.8 \| 34.8 \| 45.0 \| 56.3 \| 75.8 \| \| -1.6 \| 20.8 \| 34.8 \| 45.0 \| 56.3 \| 75.5 \| \| -1.5 \| 20.8 \| 34.8 \| 45.0 \| 56.0 \| 75.3 \| \| -1.4 \| 20.8 \| 34.8 \| 44.8 \| 56.0 \| 75.3 \| \| -1.3 \| 20.8 \| 34.5 \| 44.8 \| 55.8 \| 75.0 \| \| -1.2 \| 20.8 \| 34.5 \| 44.5 \| 55.8 \| 74.8 \| \| -1.1 \| 20.5 \| 34.5 \| 44.5 \| 55.5 \| 74.8 \| \| -1.0 \| 20.5 \| 34.3 \| 44.5 \| 55.5 \| 74.5 \| \| -0.9 \| 20.5 \| 34.3 \| 44.3 \| 55.3 \| 74.3 \| \| -0.8 \| 20.5 \| 34.3 \| 44.3 \| 55.3 \| 74.3 \| \| -0.7 \| 20.5 \| 34.0 \| 44.0 \| 55.0 \| 74.0 \| \| -0.6 \| 20.5 \| 34.0 \| 44.0 \| 55.0 \| 73.8 \| \| -0.5 \| 20.3 \| 34.0 \| 44.0 \| 54.8 \| 73.8 \| \| -0.4 \| 20.3 \| 34.0 \| 43.8 \| 54.8 \| 73.5 \| \| -0.3 \| 20.3 \| 33.8 \| 43.8 \| 54.5 \| 73.5 \| \| -0.2 \| 20.3 \| 33.8 \| 43.8 \| 54.5 \| 73.3 \| \| -0.1 \| 20.3 \| 33.8 \| 43.5 \| 54.3 \| 73.0 \| \| 0.0 \| 20.0 \| 33.5 \| 43.5 \| 54.3 \| 73.0 \| \| 0.1 \| 20.0 \| 33.5 \| 43.3 \| 54.0 \| 72.8 \| \| 0.2 \| 20.0 \| 33.5 \| 43.3 \| 54.0 \| 72.5 \| \| 0.3 \| 20.0 \| 33.3 \| 43.3 \| 53.8 \| 72.5 \| \| 0.4 \| 20.0 \| 33.3 \| 43.0 \| 53.8 \| 72.3 \| \| 0.5 \| 20.0 \| 33.3 \| 43.0 \| 53.5 \| 72.0 \| \| **Percentiles for girls** \| \| \| \| \| \| \| **BMI z-score** \| **P5** \| **P25** \| **P50** \| **P75** \| **P95** \| \| 0.6 \| 19.8 \| 33.3 \| 42.8 \| 53.5 \| 72.0 \| \| 0.7 \| 19.8 \| 33.0 \| 42.8 \| 53.3 \| 71.8 \| \| 0.8 \| 19.8 \| 33.0 \| 42.8 \| 53.3 \| 71.5 \| \| 0.9 \| 19.8 \| 33.0 \| 42.5 \| 53.3 \| 71.5 \| \| 1.0 \| 19.8 \| 32.8 \| 42.5 \| 53.0 \| 71.3 \| \| 1.1 \| 19.5 \| 32.8 \| 42.5 \| 53.0 \| 71.0 \| \| 1.2 \| 19.5 \| 32.8 \| 42.3 \| 52.8 \| 71.0 \| \| 1.3 \| 19.5 \| 32.5 \| 42.3 \| 52.8 \| 70.8 \| \| 1.4 \| 19.5 \| 32.5 \| 42.0 \| 52.5 \| 70.8 \| \| 1.5 \| 19.5 \| 32.5 \| 42.0 \| 52.5 \| 70.5 \| \| 1.6 \| 19.5 \| 32.5 \| 42.0 \| 52.3 \| 70.3 \| \| 1.7 \| 19.3 \| 32.3 \| 41.8 \| 52.3 \| 70.3 \| \| 1.8 \| 19.3 \| 32.3 \| 41.8 \| 52.0 \| 70.0 \| \| 1.9 \| 19.3 \| 32.3 \| 41.5 \| 52.0 \| 69.8 \| \| 2.0 \| 19.3 \| 32.0 \| 41.5 \| 51.8 \| 69.8 \| \| 2.1 \| 19.3 \| 32.0 \| 41.5 \| 51.8 \| 69.5 \| \| 2.2 \| 19.3 \| 32.0 \| 41.3 \| 51.5 \| 69.3 \| \| 2.3 \| 19.0 \| 31.8 \| 41.3 \| 51.5 \| 69.3 \| \| 2.4 \| 19.0 \| 31.8 \| 41.0 \| 51.3 \| 69.0 \| \| 2.5 \| 19.0 \| 31.8 \| 41.0 \| 51.3 \| 68.8 \| \| 2.6 \| 19.0 \| 31.8 \| 41.0 \| 51.0 \| 68.8 \| \| 2.7 \| 19.0 \| 31.5 \| 40.8 \| 51.0 \| 68.5 \| \| 2.8 \| 18.8 \| 31.5 \| 40.8 \| 50.8 \| 68.3 \| \| 2.9 \| 18.8 \| 31.5 \| 40.8 \| 50.8 \| 68.3 \| \| 3.0 \| 18.8 \| 31.3 \| 40.5 \| 50.5 \| 68.0 \| | \| -2.0 \| 22.0 \| 35.8 \| 45.8 \| 57.0 \| 76.3 \| \| --- \| --- \| --- \| --- \| --- \| --- \| \| -1.9 \| 21.8 \| 35.8 \| 45.8 \| 57.0 \| 76.3 \| \| -1.8 \| 21.8 \| 35.8 \| 45.8 \| 57.0 \| 76.3 \| \| -1.7 \| 21.8 \| 35.8 \| 45.8 \| 57.0 \| 76.3 \| \| -1.6 \| 21.8 \| 35.8 \| 45.8 \| 56.8 \| 76.3 \| \| -1.5 \| 21.8 \| 35.5 \| 45.8 \| 56.8 \| 76.0 \| \| -1.4 \| 21.8 \| 35.5 \| 45.8 \| 56.8 \| 76.0 \| \| -1.3 \| 21.8 \| 35.5 \| 45.8 \| 56.8 \| 76.0 \| \| -1.2 \| 21.8 \| 35.5 \| 45.8 \| 56.8 \| 76.0 \| \| -1.1 \| 21.8 \| 35.5 \| 45.5 \| 56.8 \| 76.0 \| \| -1.0 \| 21.8 \| 35.5 \| 45.5 \| 56.5 \| 75.8 \| \| -0.9 \| 21.8 \| 35.5 \| 45.5 \| 56.5 \| 75.8 \| \| -0.8 \| 21.8 \| 35.5 \| 45.5 \| 56.5 \| 75.8 \| \| -0.7 \| 21.8 \| 35.5 \| 45.5 \| 56.5 \| 75.8 \| \| -0.6 \| 21.8 \| 35.5 \| 45.5 \| 56.5 \| 75.5 \| \| -0.5 \| 21.8 \| 35.3 \| 45.5 \| 56.5 \| 75.5 \| \| -0.4 \| 21.8 \| 35.3 \| 45.3 \| 56.3 \| 75.5 \| \| -0.3 \| 21.5 \| 35.3 \| 45.3 \| 56.3 \| 75.5 \| \| -0.2 \| 21.5 \| 35.3 \| 45.3 \| 56.3 \| 75.3 \| \| -0.1 \| 21.5 \| 35.3 \| 45.3 \| 56.3 \| 75.3 \| \| 0.0 \| 21.5 \| 35.3 \| 45.3 \| 56.3 \| 75.3 \| \| 0.1 \| 21.5 \| 35.3 \| 45.3 \| 56.0 \| 75.3 \| \| 0.2 \| 21.5 \| 35.0 \| 45.0 \| 56.0 \| 75.0 \| \| 0.3 \| 21.5 \| 35.0 \| 45.0 \| 56.0 \| 75.0 \| \| 0.4 \| 21.5 \| 35.0 \| 45.0 \| 56.0 \| 75.0 \| \| 0.5 \| 21.5 \| 35.0 \| 45.0 \| 55.8 \| 74.8 \| \| **Percentiles for boys** \| \| \| \| \| \| \| **BMI z-score** \| **P5** \| **P25** \| **P50** \| **P75** \| **P95** \| \| 0.6 \| 21.5 \| 35.0 \| 44.8 \| 55.8 \| 74.8 \| \| 0.7 \| 21.3 \| 35.0 \| 44.8 \| 55.8 \| 74.5 \| \| 0.8 \| 21.3 \| 34.8 \| 44.8 \| 55.5 \| 74.5 \| \| 0.9 \| 21.3 \| 34.8 \| 44.8 \| 55.5 \| 74.3 \| \| 1.0 \| 21.3 \| 34.8 \| 44.5 \| 55.5 \| 74.3 \| \| 1.1 \| 21.3 \| 34.8 \| 44.5 \| 55.3 \| 74.0 \| \| 1.2 \| 21.3 \| 34.5 \| 44.5 \| 55.3 \| 74.0 \| \| 1.3 \| 21.3 \| 34.5 \| 44.3 \| 55.0 \| 73.8 \| \| 1.4 \| 21.0 \| 34.5 \| 44.3 \| 55.0 \| 73.5 \| \| 1.5 \| 21.0 \| 34.3 \| 44.0 \| 54.8 \| 73.5 \| \| 1.6 \| 21.0 \| 34.3 \| 44.0 \| 54.8 \| 73.3 \| \| 1.7 \| 21.0 \| 34.3 \| 44.0 \| 54.5 \| 73.0 \| \| 1.8 \| 21.0 \| 34.0 \| 43.8 \| 54.5 \| 73.0 \| \| 1.9 \| 20.8 \| 34.0 \| 43. \| 54.3 \| 72.8 \| \| 2.0 \| 20.8 \| 34.0 \| 43.5 \| 54.3 \| 72.5 \| \| 2.1 \| 20.8 \| 33.8 \| 43.5 \| 54.0 \| 72.5 \| \| 2.2 \| 20.8 \| 33.8 \| 43.5 \| 54.0 \| 72.3 \| \| 2.3 \| 20.8 \| 33.8 \| 43.3 \| 53.8 \| 72.0 \| \| 2.4 \| 20.5 \| 33.5 \| 43.3 \| 53.8 \| 71.8 \| \| 2.5 \| 20.5 \| 33.5 \| 43.0 \| 53.5 \| 71.8 \| \| 2.6 \| 20.5 \| 33.5 \| 43.0 \| 53.3 \| 71.5 \| \| 2.7 \| 20.5 \| 33.3 \| 42.8 \| 53.3 \| 71.3 \| \| 2.8 \| 20.5 \| 33.3 \| 42.8 \| 53.0 \| 71.0 \| \| 2.9 \| 20.3 \| 33.3 \| 42.5 \| 53.0 \| 71.0 \| \| 3.0 \| 20.3 \| 33.0 \| 42.5 \| 52.8 \| 70.8 \| |

*Cole & Lobstein 2012

**Supplementary Table 5: Sex-specific percentiles (P) of 25-hydroxyvitamin D (nmol/l) in girls and boys depending on usual vitamin D intake**

| **Percentiles for girls** | **Percentiles for boys** |
| --- | --- |
| \| **Vitamin D intake (µg/d)** \| **P5** \| **P25** \| **P50** \| **P75** \| **P95** \| \| --- \| --- \| --- \| --- \| --- \| --- \| | \| **Vitamin D intake (µg/d)** \| **P5** \| **P25** \| **P50** \| **P75** \| **P95** \| \| --- \| --- \| --- \| --- \| --- \| --- \| |
| \| 1.0 \| 18.0 \| 30.0 \| 39.0 \| 48.5 \| 64.5 \| \| --- \| --- \| --- \| --- \| --- \| --- \| \| 1.1 \| 18.3 \| 30.5 \| 39.3 \| 49.0 \| 65.3 \| \| 1.2 \| 18.5 \| 30.8 \| 39.8 \| 49.5 \| 66.0 \| \| 1.3 \| 18.5 \| 31.0 \| 40.3 \| 50.0 \| 66.8 \| \| 1.4 \| 18.8 \| 31.5 \| 40.8 \| 50.8 \| 67.5 \| \| 1.5 \| 19.0 \| 31.8 \| 41.3 \| 51.3 \| 68.3 \| \| 1.6 \| 19.3 \| 32.3 \| 41.5 \| 51.8 \| 69.0 \| \| 1.7 \| 19.5 \| 32.5 \| 42.0 \| 52.3 \| 69.8 \| \| 1.8 \| 19.8 \| 32.8 \| 42.5 \| 53.0 \| 70.5 \| \| 1.9 \| 19.8 \| 33.3 \| 43.0 \| 53.5 \| 71.3 \| \| 2.0 \| 20.0 \| 33.5 \| 43.5 \| 54.0 \| 72.0 \| \| 2.1 \| 20.3 \| 34.0 \| 43.8 \| 54.5 \| 72.8 \| \| 2.2 \| 20.5 \| 34.3 \| 44.3 \| 55.3 \| 73.5 \| \| 2.3 \| 20.8 \| 34.5 \| 44.8 \| 55.8 \| 74.3 \| \| 2.4 \| 21.0 \| 35.0 \| 45.3 \| 56.3 \| 75.0 \| \| 2.5 \| 21.0 \| 35.3 \| 45.8 \| 56.8 \| 75.8 \| \| 2.6 \| 21.3 \| 35.8 \| 46.0 \| 57.5 \| 76.5 \| \| 2.7 \| 21.5 \| 36.0 \| 46.5 \| 58.0 \| 77.3 \| | \| 1.0 \| 20.3 \| 33.0 \| 42.5 \| 52.8 \| 70.0 \| \| --- \| --- \| --- \| --- \| --- \| --- \| \| 1.1 \| 20.3 \| 33.0 \| 42.8 \| 53.0 \| 70.3 \| \| 1.2 \| 20.5 \| 33.3 \| 43.0 \| 53.3 \| 70.8 \| \| 1.3 \| 20.5 \| 33.5 \| 43.0 \| 53.8 \| 71.0 \| \| 1.4 \| 20.5 \| 33.5 \| 43.3 \| 54.0 \| 71.5 \| \| 1.5 \| 20.8 \| 33.8 \| 43.5 \| 54.3 \| 71.8 \| \| 1.6 \| 20.8 \| 34.0 \| 43.8 \| 54.5 \| 72.3 \| \| 1.7 \| 21.0 \| 34.3 \| 44.0 \| 54.8 \| 72.5 \| \| 1.8 \| 21.0 \| 34.3 \| 44.3 \| 55.0 \| 73.0 \| \| 1.9 \| 21.3 \| 34.5 \| 44.5 \| 55.3 \| 73.3 \| \| 2.0 \| 21.3 \| 34.8 \| 44.8 \| 55.5 \| 73.8 \| \| 2.1 \| 21.5 \| 34.8 \| 45.0 \| 56.0 \| 74.0 \| \| 2.2 \| 21.5 \| 35.0 \| 45.3 \| 56.3 \| 74.5 \| \| 2.3 \| 21.5 \| 35.3 \| 45.5 \| 56.5 \| 74.8 \| \| 2.4 \| 21.8 \| 35.3 \| 45.5 \| 56.8 \| 75.3 \| \| 2.5 \| 21.8 \| 35.5 \| 45.8 \| 57.0 \| 75.5 \| \| 2.6 \| 22.0 \| 35.8 \| 46.0 \| 57.3 \| 76.0 \| \| 2.7 \| 22.0 \| 36.0 \| 46.3 \| 57.5 \| 76.3 \| |

**Supplementary Table 6: Sex-specific percentiles (P) of 25-hydroxyvitamin D (nmol/l) in girls and boys depending on time spent outdoors**

| **Percentiles for girls** | **Percentiles for boys** |
| --- | --- |
| \| **Time spent outdoors (h)** \| **P5** \| **P25** \| **P50** \| **P75** \| **P95** \| \| --- \| --- \| --- \| --- \| --- \| --- \| | \| **Time spent outdoors (h)** \| **P5** \| **P25** \| **P50** \| **P75** \| **P95** \| \| --- \| --- \| --- \| --- \| --- \| --- \| |
| \| 0.0 \| 16.8 \| 31.0 \| 40.5 \| 50.5 \| 71.3 \| \| --- \| --- \| --- \| --- \| --- \| --- \| \| 0.1 \| 17.0 \| 31.3 \| 40.5 \| 50.8 \| 71.0 \| \| 0.2 \| 17.3 \| 31.5 \| 40.8 \| 51.0 \| 70.8 \| \| 0.3 \| 17.5 \| 31.5 \| 41.0 \| 51.0 \| 70.8 \| \| 0.4 \| 17.8 \| 31.8 \| 41.0 \| 51.3 \| 70.8 \| \| 0.5 \| 18.0 \| 31.8 \| 41.3 \| 51.3 \| 70.5 \| \| 0.6 \| 18.3 \| 32.0 \| 41.3 \| 51.5 \| 70.5 \| \| 0.7 \| 18.3 \| 32.3 \| 41.5 \| 51.8 \| 70.5 \| \| 0.8 \| 18.5 \| 32.3 \| 41.8 \| 51.8 \| 70.5 \| \| 0.9 \| 18.8 \| 32.5 \| 41.8 \| 52.0 \| 70.5 \| \| 1.0 \| 19.0 \| 32.5 \| 42.0 \| 52.3 \| 70.5 \| \| 1.1 \| 19.3 \| 32.8 \| 42.3 \| 52.3 \| 70.5 \| \| 1.2 \| 19.5 \| 32.8 \| 42.3 \| 52.5 \| 70.5 \| \| 1.3 \| 19.5 \| 33.0 \| 42.5 \| 52.8 \| 70.8 \| \| 1.4 \| 19.8 \| 33.3 \| 42.5 \| 52.8 \| 70.8 \| \| 1.5 \| 20.0 \| 33.3 \| 42.8 \| 53.0 \| 70.8 \| \| 1.6 \| 20.0 \| 33.5 \| 43.0 \| 53.3 \| 71.0 \| \| 1.7 \| 20.3 \| 33.5 \| 43.0 \| 53.5 \| 71.0 \| \| 1.8 \| 20.5 \| 33.8 \| 43.3 \| 53.5 \| 71.3 \| \| 1.9 \| 20.5 \| 33.8 \| 43.5 \| 53.8 \| 71.3 \| \| 2.0 \| 20.8 \| 34.0 \| 43.5 \| 54.0 \| 71.5 \| \| 2.1 \| 21.0 \| 34.0 \| 43.8 \| 54.0 \| 71.5 \| \| 2.2 \| 21.0 \| 34.3 \| 44.0 \| 54.3 \| 71.8 \| \| 2.3 \| 21.3 \| 34.5 \| 44.0 \| 54.5 \| 71.8 \| \| 2.4 \| 21.3 \| 34.5 \| 44.3 \| 54.8 \| 72.0 \| \| **Percentiles for girls** \| \| \| \| \| \| \| **Time spent outdoors (h)** \| **P5** \| **P25** \| **P50** \| **P75** \| **P95** \| \| 2.5 \| 21.5 \| 34.8 \| 44.3 \| 54.8 \| 72.3 \| \| 2.6 \| 21.8 \| 34.8 \| 44.5 \| 55.0 \| 72.3 \| \| 2.7 \| 21.8 \| 35.0 \| 44.8 \| 55.3 \| 72.5 \| \| 2.8 \| 22.0 \| 35.0 \| 44.8 \| 55.5 \| 72.8 \| \| 2.9 \| 22.0 \| 35.3 \| 45.0 \| 55.5 \| 72.8 \| \| 3.0 \| 22.3 \| 35.3 \| 45.3 \| 55.8 \| 73.0 \| \| 3.1 \| 22.3 \| 35.5 \| 45.3 \| 56.0 \| 73.3 \| \| 3.2 \| 22.5 \| 35.5 \| 45.5 \| 56.3 \| 73.5 \| \| 3.3 \| 22.5 \| 35.8 \| 45.8 \| 56.3 \| 73.5 \| \| 3.4 \| 22.8 \| 35.8 \| 45.8 \| 56.5 \| 73.8 \| \| 3.5 \| 22.8 \| 36.0 \| 46.0 \| 56.8 \| 74.0 \| \| 3.6 \| 22.8 \| 36.0 \| 46.0 \| 57.0 \| 74.3 \| \| 3.7 \| 23.0 \| 36.3 \| 46.3 \| 57.0 \| 74.5 \| \| 3.8 \| 23.0 \| 36.5 \| 46.5 \| 57.3 \| 74.8 \| \| 3.9 \| 23.3 \| 36.5 \| 46.5 \| 57.5 \| 74.8 \| \| 4.0 \| 23.3 \| 36.8 \| 46.8 \| 57.8 \| 75.0 \| \| 4.1 \| 23.5 \| 36.8 \| 47.0 \| 57.8 \| 75.3 \| \| 4.2 \| 23.5 \| 37.0 \| 47.0 \| 58.0 \| 75.5 \| \| 4.3 \| 23.5 \| 37.0 \| 47.3 \| 58.3 \| 75.8 \| \| 4.4 \| 23.8 \| 37.3 \| 47.5 \| 58.5 \| 76.0 \| \| 4.5 \| 23.8 \| 37.3 \| 47.5 \| 58.5 \| 76.3 \| \| 4.6 \| 24.0 \| 37.5 \| 47.8 \| 58.8 \| 76.5 \| \| 4.7 \| 24.0 \| 37.5 \| 48.0 \| 59.0 \| 76.8 \| \| 4.8 \| 24.3 \| 37.8 \| 48.0 \| 59.3 \| 76.8 \| \| 4.9 \| 24.3 \| 37.8 \| 48.3 \| 59.5 \| 77.0 \| \| 5.0 \| 24.3 \| 38.0 \| 48.3 \| 59.5 \| 77.3 \| | \| 0.0 \| 20.0 \| 32.3 \| 41.0 \| 50.8 \| 67.3 \| \| --- \| --- \| --- \| --- \| --- \| --- \| \| 0.1 \| 20.3 \| 32.5 \| 41.3 \| 51.0 \| 67.8 \| \| 0.2 \| 20.3 \| 32.5 \| 41.5 \| 51.3 \| 68.0 \| \| 0.3 \| 20.3 \| 32.8 \| 41.8 \| 51.5 \| 68.5 \| \| 0.4 \| 20.5 \| 33.0 \| 42.0 \| 51.8 \| 68.8 \| \| 0.5 \| 20.5 \| 33.3 \| 42.3 \| 52.0 \| 69.3 \| \| 0.6 \| 20.8 \| 33.3 \| 42.5 \| 52.5 \| 69.5 \| \| 0.7 \| 20.8 \| 33.5 \| 42.8 \| 52.8 \| 70.0 \| \| 0.8 \| 21.0 \| 33.8 \| 43.0 \| 53.0 \| 70.3 \| \| 0.9 \| 21.0 \| 33.8 \| 43.3 \| 53.3 \| 70.8 \| \| 1.0 \| 21.0 \| 34.0 \| 43.3 \| 53.5 \| 71.0 \| \| 1.1 \| 21.3 \| 34.3 \| 43.5 \| 53.8 \| 71.5 \| \| 1.2 \| 21.3 \| 34.5 \| 43.8 \| 54.0 \| 71.8 \| \| 1.3 \| 21.5 \| 34.5 \| 44.0 \| 54.3 \| 72.3 \| \| 1.4 \| 21.5 \| 34.8 \| 44.3 \| 54.5 \| 72.5 \| \| 1.5 \| 21.8 \| 35.0 \| 44.5 \| 55.0 \| 73.0 \| \| 1.6 \| 21.8 \| 35.0 \| 44.8 \| 55.3 \| 73.3 \| \| 1.7 \| 21.8 \| 35.3 \| 45.0 \| 55.5 \| 73.5 \| \| 1.8 \| 22.0 \| 35.5 \| 45.0 \| 55.8 \| 74.0 \| \| 1.9 \| 22.0 \| 35.5 \| 45.3 \| 56.0 \| 74.3 \| \| 2.0 \| 22.3 \| 35.8 \| 45.5 \| 56.3 \| 74.5 \| \| 2.1 \| 22.3 \| 35.8 \| 45.8 \| 56.5 \| 75.0 \| \| 2.2 \| 22.3 \| 36.0 \| 46.0 \| 56.8 \| 75.3 \| \| 2.3 \| 22.5 \| 36.3 \| 46.0 \| 56.8 \| 75.5 \| \| 2.4 \| 22.5 \| 36.3 \| 46.3 \| 57.0 \| 75.8 \| \| **Percentiles for boys** \| \| \| \| \| \| \| **Time spent outdoors (h)** \| **P5** \| **P25** \| **P50** \| **P75** \| **P95** \| \| 2.5 \| 22.5 \| 36.5 \| 46.5 \| 57.3 \| 76.0 \| \| 2.6 \| 22.8 \| 36.5 \| 46.5 \| 57.5 \| 76.5 \| \| 2.7 \| 22.8 \| 36.8 \| 46.8 \| 57.8 \| 76.8 \| \| 2.8 \| 22.8 \| 36.8 \| 47.0 \| 58.0 \| 77.0 \| \| 2.9 \| 23.0 \| 37.0 \| 47.0 \| 58.0 \| 77.3 \| \| 3.0 \| 23.0 \| 37.0 \| 47.3 \| 58.3 \| 77.5 \| \| 3.1 \| 23.0 \| 37.3 \| 47.3 \| 58.5 \| 77.5 \| \| 3.2 \| 23.3 \| 37.3 \| 47.5 \| 58.5 \| 77.8 \| \| 3.3 \| 23.3 \| 37.3 \| 47.5 \| 58.8 \| 78.0 \| \| 3.4 \| 23.3 \| 37.5 \| 47.8 \| 59.0 \| 78.3 \| \| 3.5 \| 23.3 \| 37.5 \| 47.8 \| 59.0 \| 78.5 \| \| 3.6 \| 23.3 \| 37.8 \| 48.0 \| 59.3 \| 78.5 \| \| 3.7 \| 23.5 \| 37.8 \| 48.0 \| 59.3 \| 78.8 \| \| 3.8 \| 23.5 \| 37.8 \| 48.3 \| 59.5 \| 79.0 \| \| 3.9 \| 23.5 \| 37.8 \| 48.3 \| 59.5 \| 79.0 \| \| 4.0 \| 23.5 \| 38.0 \| 48.3 \| 59.8 \| 79.3 \| \| 4.1 \| 23.5 \| 38.0 \| 48.5 \| 59.8 \| 79.5 \| \| 4.2 \| 23.8 \| 38.0 \| 48.5 \| 59.8 \| 79.5 \| \| 4.3 \| 23.8 \| 38.3 \| 48.5 \| 60.0 \| 79.8 \| \| 4.4 \| 23.8 \| 38.3 \| 48.8 \| 60.0 \| 79.8 \| \| 4.5 \| 23.8 \| 38.3 \| 48.8 \| 60.3 \| 79.8 \| \| 4.6 \| 23.8 \| 38.3 \| 48.8 \| 60.3 \| 80.0 \| \| 4.7 \| 23.8 \| 38.3 \| 48.8 \| 60.3 \| 80.0 \| \| 4.8 \| 23.8 \| 38.3 \| 49.0 \| 60.3 \| 80.3 \| \| 4.9 \| 23.8 \| 38.5 \| 49.0 \| 60.5 \| 80.3 \| \| 5.0 \| 23.8 \| 38.5 \| 49.0 \| 60.5 \| 80.3 \| |

**Supplementary Table 7: Sex-specific percentiles (P) of 25-hydroxyvitamin D (nmol/l) in girls and boys depending on cloud-modified vitamin-D UV dose (kJ/m^2^, UVDVC)**

| **Percentiles for girls** | **Percentiles for boys** |
| --- | --- |
| \| **UVDVC** \| **P5** \| **P25** \| **P50** \| **P75** \| **P95** \| \| --- \| --- \| --- \| --- \| --- \| --- \| | \| **UVDVC** \| **P5** \| **P25** \| **P50** \| **P75** \| **P95** \| \| --- \| --- \| --- \| --- \| --- \| --- \| |
| \| 0,0 \| 18,0 \| 28,8 \| 36,5 \| 44,8 \| 60,0 \| \| --- \| --- \| --- \| --- \| --- \| --- \| \| 0,1 \| 18,3 \| 29,3 \| 37,3 \| 45,8 \| 61,3 \| \| 0,2 \| 18,8 \| 30,0 \| 38,0 \| 46,8 \| 62,5 \| \| 0,3 \| 19,0 \| 30,5 \| 38,5 \| 47,5 \| 63,5 \| \| 0,4 \| 19,3 \| 31,0 \| 39,3 \| 48,3 \| 64,5 \| \| 0,5 \| 19,8 \| 31,3 \| 39,8 \| 49,0 \| 65,5 \| \| 0,6 \| 19,8 \| 31,8 \| 40,3 \| 49,5 \| 66,3 \| \| 0,7 \| 20,0 \| 32,0 \| 40,8 \| 50,0 \| 67,0 \| \| 0,8 \| 20,3 \| 32,3 \| 41,0 \| 50,5 \| 67,5 \| \| 0,9 \| 20,5 \| 32,5 \| 41,3 \| 51,0 \| 68,0 \| \| 1,0 \| 20,5 \| 32,8 \| 41,5 \| 51,3 \| 68,5 \| \| 1,1 \| 20,8 \| 33,0 \| 41,8 \| 51,5 \| 69,0 \| \| 1,2 \| 20,8 \| 33,3 \| 42,0 \| 51,8 \| 69,3 \| \| 1,3 \| 21,0 \| 33,3 \| 42,3 \| 52,0 \| 69,8 \| \| 1,4 \| 21,0 \| 33,5 \| 42,5 \| 52,3 \| 70,0 \| \| 1,5 \| 21,0 \| 33,8 \| 42,8 \| 52,5 \| 70,3 \| \| 1,6 \| 21,3 \| 33,8 \| 42,8 \| 52,8 \| 70,5 \| \| 1,7 \| 21,3 \| 34,0 \| 43,0 \| 53,0 \| 71,0 \| \| 1,8 \| 21,3 \| 34,0 \| 43,3 \| 53,3 \| 71,3 \| \| 1,9 \| 21,5 \| 34,3 \| 43,5 \| 53,5 \| 71,5 \| \| 2,0 \| 21,5 \| 34,5 \| 43,8 \| 53,8 \| 72,0 \| \| 2,1 \| 21,8 \| 34,8 \| 44,0 \| 54,3 \| 72,5 \| \| 2,2 \| 22,0 \| 35,0 \| 44,5 \| 54,8 \| 73,3 \| \| 2,3 \| 22,3 \| 35,5 \| 45,0 \| 55,5 \| 74,3 \| \| 2,4 \| 22,5 \| 36,0 \| 45,8 \| 56,3 \| 75,3 \| \| 2,5 \| 22,8 \| 36,5 \| 46,3 \| 57,0 \| 76,3 \| \| 2,6 \| 23,3 \| 37,0 \| 47,0 \| 57,8 \| 77,3 \| \| **Percentiles for girls** \| \| \| \| \| \| \| **UVDVC** \| **P5** \| **P25** \| **P50** \| **P75** \| **P95** \| \| 2,7 \| 23,5 \| 37,5 \| 47,5 \| 58,5 \| 78,0 \| \| 2,8 \| 23,8 \| 37,8 \| 48,0 \| 59,0 \| 79,0 \| \| 2,9 \| 24,0 \| 38,3 \| 48,3 \| 59,5 \| 79,5 \| \| 3,0 \| 24,0 \| 38,3 \| 48,8 \| 60,0 \| 80,0 \| \| 3,1 \| 24,0 \| 38,5 \| 48,8 \| 60,0 \| 80,3 \| \| 3,2 \| 24,3 \| 38,5 \| 48,8 \| 60,3 \| 80,5 \| \| 3,3 \| 24,3 \| 38,5 \| 49,0 \| 60,3 \| 80,5 \| \| 3,4 \| 24,3 \| 38,5 \| 49,0 \| 60,3 \| 80,5 \| \| 3,5 \| 24,3 \| 38,5 \| 49,0 \| 60,3 \| 80,5 \| \| 3,6 \| 24,3 \| 38,5 \| 49,0 \| 60,3 \| 80,5 \| \| 3,7 \| 24,3 \| 38,5 \| 49,0 \| 60,3 \| 80,5 \| \| 3,8 \| 24,3 \| 38,5 \| 49,0 \| 60,3 \| 80,5 \| \| 3,9 \| 24,3 \| 38,5 \| 49,0 \| 60,3 \| 80,5 \| \| 4,0 \| 24,3 \| 38,5 \| 49,0 \| 60,3 \| 80,5 \| \| 4,1 \| 24,3 \| 38,5 \| 49,0 \| 60,3 \| 80,5 \| \| 4,2 \| 24,3 \| 38,8 \| 49,0 \| 60,5 \| 80,8 \| \| 4,3 \| 24,3 \| 38,8 \| 49,3 \| 60,5 \| 81,0 \| \| 4,4 \| 24,5 \| 39,0 \| 49,3 \| 60,8 \| 81,3 \| \| 4,5 \| 24,5 \| 39,0 \| 49,5 \| 61,0 \| 81,8 \| \| 4,6 \| 24,8 \| 39,3 \| 50,0 \| 61,5 \| 82,3 \| \| 4,7 \| 24,8 \| 39,8 \| 50,3 \| 62,0 \| 82,8 \| \| 4,8 \| 25,0 \| 40,0 \| 50,8 \| 62,5 \| 83,8 \| \| 4,9 \| 25,3 \| 40,5 \| 51,3 \| 63,3 \| 84,5 \| \| 5,0 \| 25,5 \| 41,0 \| 51,8 \| 64,0 \| 85,5 \| \| 5,1 \| 26,0 \| 41,3 \| 52,5 \| 64,5 \| 86,3 \| \| 5,2 \| 26,3 \| 41,8 \| 53,0 \| 65,3 \| 87,3 \| \| 5,3 \| 26,5 \| 42,3 \| 53,5 \| 66,0 \| 88,0 \| \| 5,4 \| 26,8 \| 42,5 \| 54,0 \| 66,5 \| 88,8 \| \| 5,5 \| 27,0 \| 43,0 \| 54,5 \| 67,0 \| 89,5 \| | \| 0,0 \| 20,0 \| 31,0 \| 39,3 \| 48,0 \| 63,0 \| \| --- \| --- \| --- \| --- \| --- \| --- \| \| 0,1 \| 20,3 \| 31,3 \| 39,5 \| 48,5 \| 63,5 \| \| 0,2 \| 20,3 \| 31,5 \| 39,8 \| 48,8 \| 64,0 \| \| 0,3 \| 20,5 \| 31,8 \| 40,3 \| 49,3 \| 64,8 \| \| 0,4 \| 20,8 \| 32,0 \| 40,5 \| 49,5 \| 65,3 \| \| 0,5 \| 20,8 \| 32,3 \| 40,8 \| 50,0 \| 65,8 \| \| 0,6 \| 21,0 \| 32,8 \| 41,3 \| 50,5 \| 66,3 \| \| 0,7 \| 21,3 \| 33,0 \| 41,5 \| 50,8 \| 66,8 \| \| 0,8 \| 21,3 \| 33,3 \| 41,8 \| 51,3 \| 67,3 \| \| 0,9 \| 21,5 \| 33,5 \| 42,0 \| 51,5 \| 67,8 \| \| 1,0 \| 21,8 \| 33,8 \| 42,5 \| 52,0 \| 68,3 \| \| 1,1 \| 21,8 \| 34,0 \| 42,8 \| 52,5 \| 68,8 \| \| 1,2 \| 22,0 \| 34,3 \| 43,0 \| 52,8 \| 69,3 \| \| 1,3 \| 22,3 \| 34,5 \| 43,5 \| 53,3 \| 69,8 \| \| 1,4 \| 22,3 \| 34,8 \| 43,8 \| 53,5 \| 70,3 \| \| 1,5 \| 22,5 \| 35,0 \| 44,0 \| 54,0 \| 70,8 \| \| 1,6 \| 22,8 \| 35,3 \| 44,5 \| 54,3 \| 71,5 \| \| 1,7 \| 22,8 \| 35,5 \| 44,8 \| 54,8 \| 72,0 \| \| 1,8 \| 23,0 \| 35,8 \| 45,0 \| 55,3 \| 72,5 \| \| 1,9 \| 23,3 \| 36,0 \| 45,3 \| 55,5 \| 73,0 \| \| 2,0 \| 23,3 \| 36,3 \| 45,8 \| 56,0 \| 73,5 \| \| 2,1 \| 23,5 \| 36,5 \| 46,0 \| 56,3 \| 74,0 \| \| 2,2 \| 23,8 \| 36,8 \| 46,3 \| 56,8 \| 74,5 \| \| 2,3 \| 23,8 \| 37,0 \| 46,8 \| 57,3 \| 75,0 \| \| 2,4 \| 24,0 \| 37,3 \| 47,0 \| 57,5 \| 75,5 \| \| 2,5 \| 24,3 \| 37,5 \| 47,3 \| 58,0 \| 76,0 \| \| 2,6 \| 24,3 \| 37,8 \| 47,5 \| 58,3 \| 76,5 \| \| **Percentiles for boys** \| \| \| \| \| \| \| **UVDVC** \| **P5** \| **P25** \| **P50** \| **P75** \| **P95** \| \| 2,7 \| 24,5 \| 38,0 \| 48,0 \| 58,8 \| 77,0 \| \| 2,8 \| 24,8 \| 38,3 \| 48,3 \| 59,0 \| 77,8 \| \| 2,9 \| 24,8 \| 38,5 \| 48,5 \| 59,5 \| 78,3 \| \| 3,0 \| 25,0 \| 38,8 \| 49,0 \| 60,0 \| 78,8 \| \| 3,1 \| 25,3 \| 39,0 \| 49,3 \| 60,3 \| 79,3 \| \| 3,2 \| 25,3 \| 39,3 \| 49,5 \| 60,8 \| 79,8 \| \| 3,3 \| 25,5 \| 39,5 \| 49,8 \| 61,0 \| 80,3 \| \| 3,4 \| 25,8 \| 39,8 \| 50,3 \| 61,5 \| 80,8 \| \| 3,5 \| 25,8 \| 40,0 \| 50,5 \| 62,0 \| 81,3 \| \| 3,6 \| 26,0 \| 40,3 \| 50,8 \| 62,3 \| 81,8 \| \| 3,7 \| 26,3 \| 40,5 \| 51,3 \| 62,8 \| 82,3 \| \| 3,8 \| 26,3 \| 40,8 \| 51,5 \| 63,0 \| 82,8 \| \| 3,9 \| 26,5 \| 41,0 \| 51,8 \| 63,5 \| 83,3 \| \| 4,0 \| 26,8 \| 41,3 \| 52,3 \| 64,0 \| 83,8 \| \| 4,1 \| 26,8 \| 41,5 \| 52,5 \| 64,3 \| 84,5 \| \| 4,2 \| 27,0 \| 41,8 \| 52,8 \| 64,8 \| 85,0 \| \| 4,3 \| 27,0 \| 42,0 \| 53,0 \| 65,0 \| 85,5 \| \| 4,4 \| 27,3 \| 42,3 \| 53,5 \| 65,5 \| 86,0 \| \| 4,5 \| 27,5 \| 42,8 \| 53,8 \| 65,8 \| 86,5 \| \| 4,6 \| 27,5 \| 43,0 \| 54,0 \| 66,3 \| 87,0 \| \| 4,7 \| 27,8 \| 43,3 \| 54,5 \| 66,8 \| 87,5 \| \| 4,8 \| 28,0 \| 43,5 \| 54,8 \| 67,0 \| 88,0 \| \| 4,9 \| 28,0 \| 43,8 \| 55,0 \| 67,5 \| 88,5 \| \| 5,0 \| 28,3 \| 44,0 \| 55,3 \| 67,8 \| 89,0 \| \| 5,1 \| 28,5 \| 44,3 \| 55,8 \| 68,3 \| 89,5 \| \| 5,2 \| 28,5 \| 44,5 \| 56,0 \| 68,8 \| 90,0 \| \| 5,3 \| 28,8 \| 44,8 \| 56,3 \| 69,0 \| 90,8 \| \| 5,4 \| 29,0 \| 45,0 \| 56,8 \| 69,5 \| 91,3 \| \| 5,5 \| 29,0 \| 45,3 \| 57,0 \| 69,8 \| 91,8 \| |

**Supplementary Table 8: Sex-specific percentiles (P) of 25-hydroxyvitamin D (nmol/l) in girls and boys depending on waist-to-height ratio**

| **Percentiles for girls** | **Percentiles for boys** |
| --- | --- |
| \| **Waist-to-height ratio** \| **P5** \| **P25** \| **P50** \| **P75** \| **P95** \| \| --- \| --- \| --- \| --- \| --- \| --- \| | \| **Waist-to-height ratio** \| **P5** \| **P25** \| **P50** \| **P75** \| **P95** \| \| --- \| --- \| --- \| --- \| --- \| --- \| |
| \| 0.35 \| 20.3 \| 34.0 \| 44.0 \| 54.8 \| 73.8 \| \| --- \| --- \| --- \| --- \| --- \| --- \| \| 0.36 \| 20.3 \| 34.0 \| 44.0 \| 54.8 \| 73.5 \| \| 0.37 \| 20.3 \| 34.0 \| 43.8 \| 54.8 \| 73.5 \| \| 0.38 \| 20.3 \| 33.8 \| 43.8 \| 54.5 \| 73.3 \| \| 0.39 \| 20.3 \| 33.8 \| 43.8 \| 54.5 \| 73.3 \| \| 0.40 \| 20.3 \| 33.8 \| 43.5 \| 54.3 \| 73.0 \| \| 0.41 \| 20.0 \| 33.8 \| 43.5 \| 54.3 \| 72.8 \| \| 0.42 \| 20.0 \| 33.5 \| 43.5 \| 54.3 \| 72.8 \| \| 0.43 \| 20.0 \| 33.5 \| 43.3 \| 54.0 \| 72.5 \| \| 0.44 \| 20.0 \| 33.5 \| 43.3 \| 54.0 \| 72.5 \| \| 0.45 \| 20.0 \| 33.3 \| 43.3 \| 53.8 \| 72.3 \| \| 0.46 \| 20.0 \| 33.3 \| 43.0 \| 53.8 \| 72.3 \| \| 0.47 \| 20.0 \| 33.3 \| 43.0 \| 53.5 \| 72.0 \| \| 0.48 \| 19.8 \| 33.3 \| 43.0 \| 53.5 \| 71.8 \| \| 0.49 \| 19.8 \| 33.0 \| 42.8 \| 53.5 \| 71.8 \| \| 0.50 \| 19.8 \| 33.0 \| 42.8 \| 53.3 \| 71.5 \| \| 0.51 \| 19.8 \| 33.0 \| 42.8 \| 53.3 \| 71.5 \| \| 0.52 \| 19.8 \| 33.0 \| 42.5 \| 53.0 \| 71.3 \| \| 0.53 \| 19.8 \| 32.8 \| 42.5 \| 53.0 \| 71.3 \| \| 0.54 \| 19.5 \| 32.8 \| 42.5 \| 52.8 \| 71.0 \| \| 0.55 \| 19.5 \| 32.8 \| 42.3 \| 52.8 \| 71.0 \| | \| 0.35 \| 21.0 \| 34.3 \| 44.0 \| 54.5 \| 73.0 \| \| --- \| --- \| --- \| --- \| --- \| --- \| \| 0.36 \| 21.3 \| 34.5 \| 44.3 \| 54.8 \| 73.3 \| \| 0.37 \| 21.3 \| 34.8 \| 44.5 \| 55.0 \| 73.8 \| \| 0.38 \| 21.3 \| 34.8 \| 44.5 \| 55.3 \| 74.0 \| \| 0.39 \| 21.5 \| 35.0 \| 44.8 \| 55.5 \| 74.3 \| \| 0.40 \| 21.5 \| 35.0 \| 45.0 \| 55.8 \| 74.5 \| \| 0.41 \| 21.5 \| 35.3 \| 45.0 \| 55.8 \| 74.8 \| \| 0.42 \| 21.5 \| 35.3 \| 45.3 \| 56.0 \| 75.0 \| \| 0.43 \| 21.8 \| 35.3 \| 45.3 \| 56.3 \| 75.3 \| \| 0.44 \| 21.8 \| 35.5 \| 45.3 \| 56.3 \| 75.3 \| \| 0.45 \| 21.8 \| 35.5 \| 45.5 \| 56.3 \| 75.3 \| \| 0.46 \| 21.8 \| 35.5 \| 45.5 \| 56.3 \| 75.3 \| \| 0.47 \| 21.8 \| 35.5 \| 45.3 \| 56.3 \| 75.3 \| \| 0.48 \| 21.8 \| 35.3 \| 45.3 \| 56.3 \| 75.0 \| \| 0.49 \| 21.5 \| 35.3 \| 45.3 \| 56.0 \| 75.0 \| \| 0.50 \| 21.5 \| 35.0 \| 45.0 \| 55.8 \| 74.5 \| \| 0.51 \| 21.5 \| 35.0 \| 44.8 \| 55.5 \| 74.3 \| \| 0.52 \| 21.3 \| 34.8 \| 44.5 \| 55.3 \| 74.0 \| \| 0.53 \| 21.3 \| 34.5 \| 44.3 \| 55.0 \| 73.5 \| \| 0.54 \| 21.0 \| 34.3 \| 44.0 \| 54.5 \| 73.0 \| \| 0.55 \| 21.0 \| 34.0 \| 43.8 \| 54.3 \| 72.5 \| |

| **Girls** | **Boys** |
| --- | --- |
|  |  |

**Supplementary Figure 1:** 25-Hydroxy-vitamin D (25(OH)D) percentile curves for girls and boys by waist-to-height ratio.

Gray: deficient status (<50 nmol/l), light gray: insufficient status (50-<75 nmol/l), white: sufficient status (≥75 nmol/l)

P, percentile

| **Girls (9 years, UVDVC=4): BMI z-score of 2.3** | **Girls (9 years, UVDVC=4): BMI z-score of 0** |
| --- | --- |
| 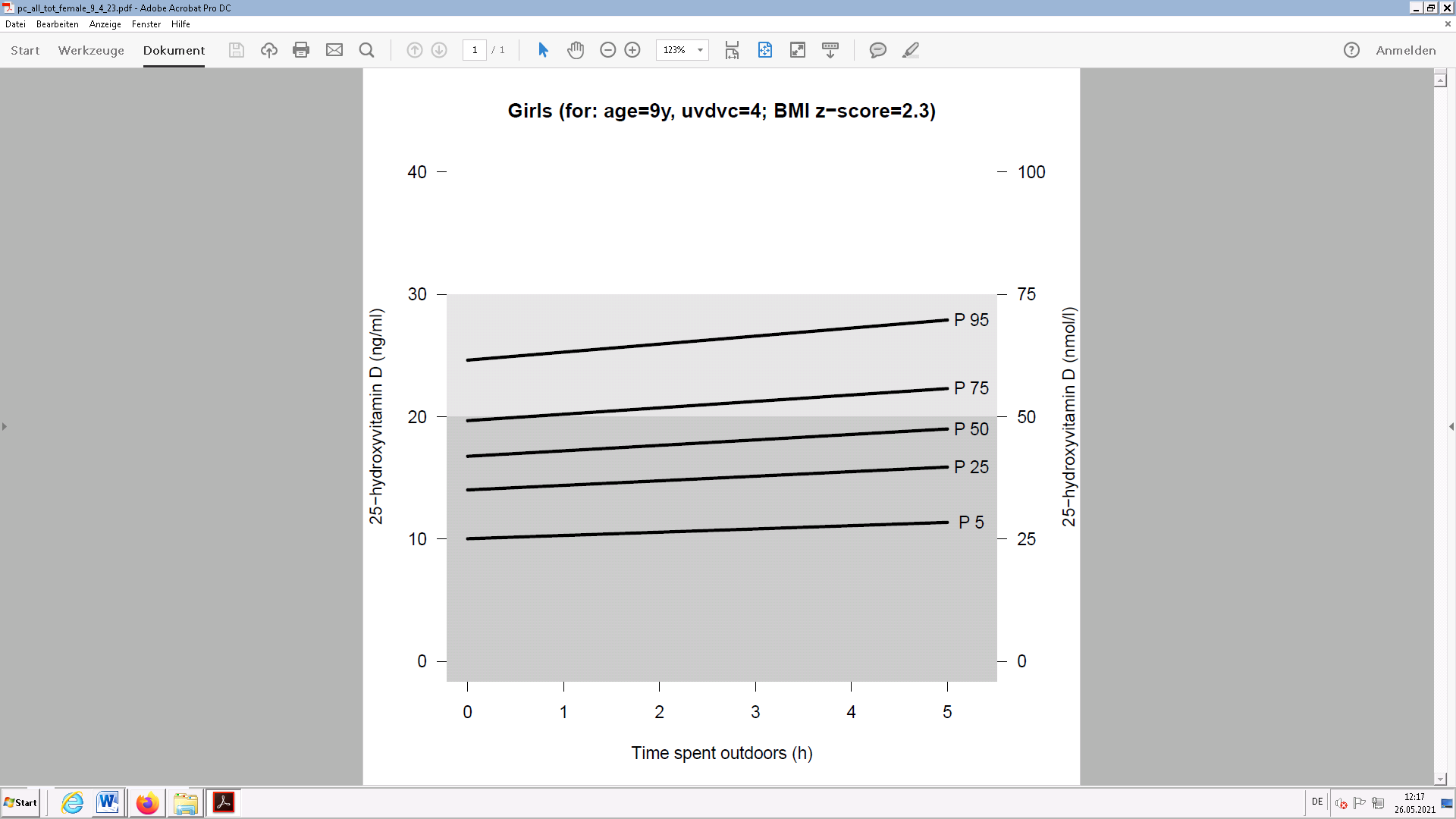 | 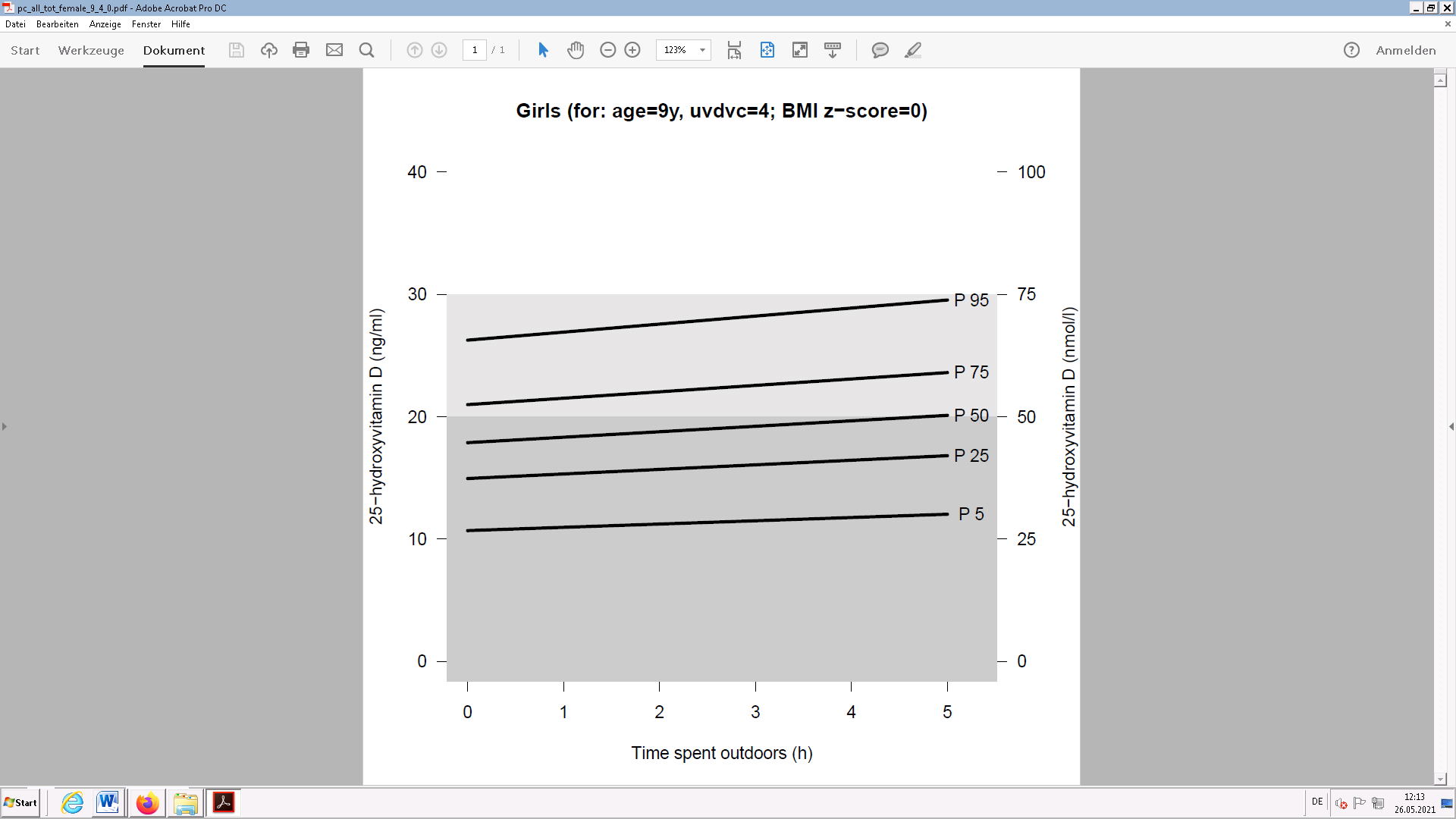 |

**Supplementary Figure 2:** 25-Hydroxyvitamin D (25(OH)D) percentile curves for girls simultaneously depending on age, UV dose (UVDVC), BMI z-score* and time spent outdoors: depicted for fix age (9.0 years), UVDVC (4) and BMI z-score* (0 (normal weight) and 2.3 (overweight)) and continuous time spent outdoors (h).

Gray: deficient status (<50 nmol/l), light gray: insufficient status (50-<75 nmol/l), white: sufficient status (≥75 nmol/l)

* Cole and Lobstein 2012

BMI, body mass index; P, percentile; UVDVC, cloud-modified vitamin-D UV dose of the second last month before blood draw
